# Trends in health behaviors over 20 years: findings from the 1998-2018 Korea National Health and Nutrition Examination Survey

**DOI:** 10.4178/epih.e2021026

**Published:** 2021-04-19

**Authors:** Soyeon Kim, Sunhye Choi, Jihee Kim, Suyeon Park, Youngtaek Kim, Ok Park, Kyungwon Oh

**Affiliations:** 1 Division of Health and Nutrition Survey and Analysis, Bureau of Chronic Disease Prevention and Control, Korea Disease Control and Prevention Agency, Cheongju, Korea; 2 Public Health Medical Service Office, Chungnam National University Hospital, Daejeon, Korea

**Keywords:** Korea National Health and Nutrition Examination Survey, Health behaviors, Cigarette smoking, Alcohol drinking, Physical activity

## Abstract

**OBJECTIVES:**

This study aimed to examine the trends in health behaviors in Korean population using data from the Korea National Health and Nutrition Examination Survey (KNHANES).

**METHODS:**

The subjects were 96,408 adults aged 19 years or older who participated in the first (1998) through seventh (2016-2018) KNHANES health interview. The prevalence of health behaviors (cigarette smoking, alcohol drinking, and physical activity) and annual percent change (APC) were estimated using SAS and the Joinpoint program.

**RESULTS:**

The prevalence of current cigarette smoking in men decreased by 2.8%p (APC=-2.8, p<0.001) annually over the 20-year period, and the prevalence of exposure to secondhand smoke at home substantially decreased compared to 2005 (APC=-8.8, p<0.001). Compared to 2005, the prevalence of current alcohol drinking in women, but not men, increased (APC=2.0, p<0.001), and the prevalence of binge drinking decreased in men (APC=-0.7, p<0.001) and increased in women (APC=2.4, p<0.001). The prevalence of aerobic physical activity decreased from 2014 in both gendersd (p<0.001). The prevalence of healthy behaviors practice (non-smoking, alcohol abstinence, and aerobic physical activity) was down-trending (APC=-5.3, p<0.001), especially among women (APC=-6.4, p<0.001).

**CONCLUSIONS:**

Over the past 20 years, smoking behaviors improved. However, drinking behavior was unchanged and physical activity indicators markedly decreased. More active programs are necessary for improving health behaviors, which are major risk factors linked to chronic diseases.

## INTRODUCTION

Death due to non-communicable disease (NCD) accounts for approximately 65% of all deaths globally [1]. Likewise, in Korea, death from NCD like cancer, diabetes, and cardiovascular disease comprises more than 80% of all deaths [2].

Health behaviors like cigarette smoking, alcohol drinking, and physical activity (PA) are closely associated with the precursors of chronic diseases, such as hypertension and dyslipidemia [3], the occurrence of chronic diseases, including cancer and cardiovascular disease (and the associated burden of disease), and early death [4,5]. In addition, smoking cessation, drinking reduction, and PA are closely linked, both independently and in combination, to health-related quality of life and healthy life expectancy [6-10]. Accordingly, most countries, including Korea, have advanced health policies in which the top priorities are health promotion and NCD prevention through the improvement of health behaviors. In National Health Plan (HP), which aims to spread healthy lifestyles, targets were established for major health indicators in the areas of smoking cessation, drinking reduction, PA, nutrition, and the gap in health equity (e.g., income level). The progress toward targets is monitored through the Korea National Health and Nutrition Examination Survey (KNHANES).

In this study, we examined trends in important health behaviors, such as cigarette smoking, alcohol drinking, and PA by utilizing the KNHANES data (1998-2018) to provide information essential to chronic disease prevention and management.

## MATERIALS AND METHODS

### Subjects

The KNHANES is conducted to estimate national statistics regarding the health and nutritional status of Korean citizens. It is composed of health examination, health interview, and nutrition survey. Since 1998, a two-stage stratified cluster sampling method was selected for about 200 primary sampling units (PSUs) and 20 households to 23 households per PSU. All eligible members aged 1 year and over within sample households become the target sample persons. The annual survey response rate was approximately 75%. The subjects included in this study were the survey participants aged 19 years or older in the first (1998) through the seventh (2016-2018) KNHANES.

### Health interview

The health interview was divided into face-to-face interview by trained interviewers (medical condition, healthcare utilization, injuries, etc.) and self-administered survey (cigarette smoking, alcohol drinking, etc.). Survey items were designed slightly differently for children (aged 1-11 years), adolescents (aged 12-18 years), and adults (aged ≥ 19 years). In this study, the analysis was limited to the cigarette smoking, alcohol drinking, and PA data collected in the self-administered section of the health interview (PA data has been collected by face-to-face interviews since 2014).

For cigarette smoking, information regarding lifetime history of smoking, current smoking status, and exposure to secondhand smoke at home, work, and public places (added 2013) were assessed. Additionally, in 2013, an instruction was added for survey participants to specifically consider the last 7 days only when responding to the questions on exposure to secondhand smoke. Starting in 2005, data on the frequency and volume of alcohol consumption and frequency of binge drinking using the Alcohol Use Disorder Identification Test (AUDIT) and data on PA using the short form of the International PA Questionnaires (IPAQ short form) began to be collected. The questionnaires consisted of a total of 7 items that assessed the amount of time spent on vigorous-intensity PA, moderate-intensity PA, walking, and sedentary time. In 2014, the Global PA Questionnaire (GPAQ) was introduced, allowing for the participation of PA to be examined in the domains of work, leisure, and transportation. The GPAQ consisted of a total of 16 items regarding the amount of time spent on moderate-intensity activity, vigorous intensity activity, and sitting in each of the three categories (work, leisure, and transportation). Since the questionnaire was difficult to answer, it was changed to face-to-face interview instead of self-administered survey. Other than slight changes to wording, the items regarding muscle strengthening did not undergo any major revisions since they were introduced in 2005.

### Definitions of health behavior indicators

Current cigarette smokers were defined as individuals with a lifetime cigarette smoking history of 5 packs or more who also currently smoked. The prevalence of exposure to secondhand smoke inside home/public places was estimated by using the number of current non-smokers as the denominator and calculating the rate of non-smokers exposed to secondhand smoke inside home/public places during the last 7 days. When calculating the prevalence of exposure to secondhand smoke in indoor workplaces, the denominator was the number of current workers classified as current non-smokers and the numerator was the number of those exposed to secondhand smoke in indoor workplaces during the last 7 days. Current alcohol drinkers were defined as individuals who consumed alcohol, on average, at least once a month, and binge drinkers as individuals who binge drank (consuming 7 and 5 glasses of alcohol in a single occasion for men and women, respectively) at least once a month on average. The prevalence of aerobic PA was estimated in accordance with the PA Guidelines for Koreans, by calculating the rate of individuals who typically performed moderate and vigorous intensity PA for a total of 150 minutes or more per week. In this calculation, work, leisure, and transportation time were combined and the amount of time spent on vigorous intensity PA was doubled. The prevalence of muscle strengthening was estimated by calculating the rate of individuals who performed muscle strengthening at least twice per week regardless of the type and duration.

Based on the definition of healthy behavior practice as the practice of engaging in aerobic PA, not smoking, and abstaining from alcohol, the prevalence of healthy behaviors could be calculated. Non-smoking was defined as a lifetime history of non-smoking or current non-smoking with a history of smoking. Alcohol abstinence was defined as not drinking or drinking alcohol less than once a month in the previous 1 year. PA was defined as practicing aerobic PA 150 minutes or more in a typical week. To calculate the prevalence of aerobic PA, the data were collected using two different questionnaires, the IPAQ from 2007-2013 and the GPAQ starting in 2014.

### Statistical analysis

All analyses were performed using SAS version 9.4 (SAS Institute Inc., Cary, NC, USA) and Joinpoint Regression Program version 4.1.1.1 (US National Cancer Institute [NCI], Bethesda, MD, USA). To represent the Korean population, the sampling weights assigned to participants were applied to all analyses. Sampling weights were generated by considering complex sample design, non-response rate of the target population, and post-stratification. To adjust differences in results from change in age structure of each year, age-standardized prevalence was calculated using the age- and gender-specific structures of estimated population based on the 2005 population projections for Korea. Trends in cigarette smoking, alcohol drinking, PA prevalence by gender and household income level were age-standardized prevalence and were calculated using SAS (PROC SURVEYREG). The prevalence indicators by age were crude rates and were calculated using SAS (PROC SURVEYMEANS). The estimates and their standard errors obtained from SAS were input into NCI’s Joinpoint program, the model was estimated by setting the Joinpoint as 0 or 1, and the annual percent change (APC) was calculated. APC verified that the annual rate of change was “0” under the significance level of 0.05, and the Monte Carlo method in Joinpoint Regression Program was used to test the statistical significance of the optimal model.

### Ethics statement

This study was approved by the Institutional Review Board of the Korea Disease Control and Prevention Agency (formerly Korea Center for Disease Control and Prevention) (2007-2014, 2018). For certain year (2015-2017), ethical approval was waived by the Act (Article 2, Paragraph 1) and Enforcement Regulation (Article 2, Paragraph 2, item 1) of Bioethics and Safety Act.

## RESULTS

The demographic characteristics of the KNHANES participants (1998-2018) are shown in Table 1. The total number of subjects aged 19 years or older was 96,408. Of those, 42,071 were men and 54,337 were women, making women a higher proportion of the sample than men. The proportion of participants aged between 19 years and 49 years decreased, while the proportion of those aged ≥ 50 years increased over time.

The prevalence of current cigarette smoking substantially decreased in men, from 66.3% in 1998 to 36.7% in 2018 (APC=-2.8, p<0.001). Over the same time period, this prevalence, however, increased slightly in women, from 6.5% to 7.5%, but the change was not statistically significant (APC=0.6) (Table 2). In men, the prevalence of current cigarette smoking significantly decreased in every age group and income level (p<0.001), but the gap between the uppermost and lowest income levels did not decrease after 2010, maintaining approximately a difference of 10%p each year (31.0% and 40.1% in 2018, respectively). In contrast, the prevalence of current cigarette smoking in women increased in the 19-29, 30-39, and 40-49 age groups (p<0.001), while it decreased in those aged ≥ 60 years. The prevalence of current non-smokers’ exposure to secondhand smoke at home, workplace, and in public places greatly diminished, with decreases of 14.5%p for home (18.5% in 2005 to 4.0% in 2018), 25.4%p for indoor workplaces (36.9% in 2008 to 11.5% in 2018), and 41.1%p for indoor public places (58.0% in 2013 to 16.9% in 2018) (p<0.001) (Table 3).

The prevalence of current alcohol drinking did not change in men from 2005 to 2018, whereas it increased in women, from 37.0% in 2005 to 51.2% in 2018 (APC=2.0, p<0.001) (Table 4). The prevalence among women significantly increased in all age groups except for those aged ≥ 70 years (p<0.001) and in all income levels (p<0.001). The prevalence of binge drinking in men decreased from 55.3% in 2005 to 50.8% in 2018, and the extent of the decrease was particularly sizable in the 19-29 age group (APC=-1.4, p<0.001) (Table 5). In contrast, the prevalence of binge drinking in women increased from 17.2% in 2005 to 26.9% in 2018 (APC, 2.4, p<0.001), rising in all age groups except for those aged ≥ 70 years. The increase in the 19-29 age group was particularly substantial (19.1%p), increasing from 30.7% in 2005 to 49.8% in 2018. Additionally, the prevalence increased for all income levels (p<0.001).

The prevalence of aerobic PA decreased by approximately 25%p in both genders, from 76.3% to 51.0% in men between 2007 and 2018, respectively (APC=-4.2, p<0.001) and from 68.9% to 44.0% in women during the same period (APC=-4.7, p<0.001) (Table 6). The prevalence significantly decreased, especially after 2014, in both genders in every age group and every income level (p<0.001). The prevalence of muscle strengthening did not change from 2005 to 2018 in men, whereas it increased in women, from 11.2% in 2005 to 14.9% in 2018 (APC=2.3, p<0.001). The increasing trend was clear in the 60-69 and 70-79 age groups (data not shown) [11].

The prevalence of healthy behaviors practice dropped by approximately 10%p, from 25.1% in 2007 to 14.5% in 2018 (APC=-5.3, p<0.001) (Table 7). The prevalence decreased in both men and women; however, in men, it significantly decreased in those aged ≥ 50 years, while in women, the prevalence significantly decreased in all the age groups. Regarding changes in the prevalence of healthy behaviors practice, the decrease was smaller in men since cigarette smoking and alcohol drinking improved in the 19-29 and 20-29 age groups. In contrast, the prevalence among women decreased by 19.0%p compared to 2007 (APC=-6.4, p<0.001) and by 8.8%p compared to 2014, because alcohol drinking and PA deteriorated in most age groups.

## DISCUSSION

In this study, changes in major health behaviors of Korean adults were investigated using the KNHANES data. The prevalence of cigarette smoking and exposure to secondhand smoke consistently decreased, as well as the prevalence of PA. Regarding alcohol drinking, there was no considerable change in men, whereas in women, the prevalence of alcohol drinking and binge drinking both increased (Supplementary Material 1).

Awareness of the harm of smoking and the need for smoking cessation has increased through the smoking cessation movement spurred by private sector in the late 1980s [12]. In 1995, the Korean government also established legal foundations for tobacco regulations through the National Health Promotion Act and promoted policies (e.g., reinforcing legal and institutional foundations, a smoking cessation campaign and support services for smoking cessation, and expanding non-smoking areas) to ratify and implement the 2005 World Health Organization Framework Convention on Tobacco Control [13-16]. Owing to these efforts, the prevalence of current cigarette smoking among men between 2013 and 2014 was approximately 43%, which was more than 20%p less than that in 1998. Since 2015, policies, such as the increase in cigarette prices, smoking cessation campaigning in the mass media, the expansion of support services for smoking cessation, and the introduction of cigarette health warning on cigarette packs (2016) have been implemented [15-17], and in 2018, the prevalence of current cigarette smoking among men had further decreased to 36.7%. Additionally, starting in 2012, various enforcements have been implemented for the purpose of preventing harm from secondhand smoke, such as smoking prohibition in all public facilities and the expansion of indoor non-smoking areas, such as in restaurants. Consequently, the prevalence of exposure to secondhand smoke has dropped to approximately a third of what it was in 1998. However, despite the decreasing trend, the prevalence of current cigarette smoking in men is still more than twice that of other countries, such as the United States (16.9% in those aged ≥ 18 years in 2015-2017), the United Kingdom (16.5% in those aged ≥ 18 years in 2018), and Japan (17.8% in those aged ≥ 20 years in 2018) [18-20]. Since the decreasing trend in current cigarette smoking prevalence has recently slowed, the use of tobacco products other than cigarettes has increased, and there continues to be a huge gap between the current prevalence and the HP 2020 goal; more powerful policies should be implemented with continuity. In 2018, the use of heated tobacco products and e-cigarettes increased to about 5 times that of 2013, the year when the relevant survey items were introduced to the KNHANES. Additionally, the prevalence of the dual use of conventional cigarettes and vaping products (including heated tobacco products and ecigarettes) increased up to 22.5%. These findings suggest the need to investigate the harms of new tobacco products more actively and monitor use behavior to develop preemptive policies in preparation for a potential increase in prevalence due to these new products.

Unlike in men, the prevalence of current cigarette smoking in women was found to be low relative to other countries [9,10,18], but there was an increasing trend in the 19-29, 30-39, and 40-49 age groups. The increase in working women, the availability of flavored and low-nicotine cigarettes, which appeal more to women [21], and the reduction in underreporting as the social stigma of women smokers has lessened are potential factors contributing to the increase in women smokers [22]. Given that, to date, the focus of support services for smoking cessation and related policies have been on men due to their high prevalence of cigarette smoking, in-depth studies, including qualitative research on women’s smoking behavior and relevant factors, are needed and effective smoking cessation programs specifically designed for women need to be developed [23].

The prevalence of current alcohol drinking and binge drinking in men were 70% and 50%, respectively, without much change over time. Although it is difficult to make accurate between-country comparisons because the definitions of the indicators are slightly different, these rates are higher compared with other countries [24]. One possible explanation why these rates were high could be that programs have primarily been designed for education, and the screening and treatment of high-risk drinking and alcohol addiction, while access to alcohol itself had not been focused [25,26]. Currently, stronger policies are being implemented for the prevention of alcohol-induced harm, such as prohibiting alcohol consumption in public places and establishing guidelines regarding scenes of drinking in the media. Thus, changes in alcohol consumption trends should continue to be followed [27-29]. In women, the prevalence of current alcohol drinking and binge drinking both increased significantly, with considerable increases in the 19-29, 30-39, and 40-49 age groups. Like the increase in cigarette smoking, an increase in binge drinking among women may be explained by the increase in working women, increased access due to the development and availability of different types of alcohol products that appeal to women (such as low-alcohol and fruit-flavored soju products), and the decrease in negative perceptions of women drinkers [21]. In the United States, the prevalence of high-risk drinking and alcohol use disorder has increased among women in comparison to men, and it has been suggested that, like Korea, these increases may be associated with increased opportunities for women through education and work and an increased permissive to drinking in the social atmosphere [24,30,31].

The prevalence of aerobic PA consistently showed a decreasing trend. In 2018, the prevalence was 51% in men and 44% in women. The analysis conducted to examine the decreasing trend by area (work, transportation, and leisure activity) showed a 6.6%p increase between 2014 and 2018 in those not engaging in transport-related PA (38.5 and 45.1%, respectively). However, no sizable increase was noted in either the prevalence of those not performing work-related PA (97.4% in 2014 and 98.6% in 2018) or those not performing leisure-related PA (90.2% in 2014 and 91.7% in 2018). Accordingly, the decrease in the prevalence of aerobic PA seemed to be the result of reduced PA for transportation. Since PA practice is closely linked to personal, socioeconomic, and environmental factors, to increase PA, accessibility should be enhanced through providing knowledge, motivating individuals, and establishing an appropriate environment [32-35]. PA indicators have not improved, even though the importance of PA has been emphasized through the PA Guidelines for Koreans since 2013. Additionally, several individual programs to promote PA have been executed as part of the integrated health promotion program in the local community. Accordingly, it seems necessary to identify the causes of the failure by conducting in-depth analyses and developing strategies to increase PA.

The prevalence of healthy behaviors practice (non-smoking, alcohol abstinence, and aerobic PA), which was 25.1% in 2007, decreased to 14.5% in 2018, which was a decrease of 10.6%p. The prevalence was greatly reduced in women. Whereas in men, the prevalence of healthy behaviors practice increased by 5.1%p (12.3% in 2014 to 17.4% in 2018) owing to improved cigarette smoking and alcohol drinking behaviors in the 19-29 age group, women’s health behaviors deteriorated overall, which was revealed in the decreased prevalence of healthy behaviors practice in every age group and income level. Healthy behaviors, such as smoking cessation, drinking reduction, and PA have been associated with a lower risk cancer and cardiovascular disease-related death, a lower risk of overall death, and an increased healthy life expectancy [8-10,36-38]. In this study, the level of healthy behavior practice was very low. Only 1 out of 10 men and 2 out of 10 women practiced healthy behaviors, without desirable diet and healthy weight considered. This finding suggests an urgent need for policies and programs to improve individual health behaviors. It is important to note that this study aimed to identify 20-year trends in health behaviors, which are the risk factors of chronic diseases. However, while these trends were examined for smoking behavior, it was not possible to examine 20-year trends for alcohol drinking and PA because new questionnaires were added or old ones were replaced during the study period.

This KNHANES data analysis covering a span of 20 years found that, except for cigarette smoking in men, health behaviors did not improve in Korea, as shown by decreased levels of PA and increased levels of alcohol drinking and binge drinking in women. This finding is believed to be linked to the increased prevalence of chronic diseases like obesity, dyslipidemia, and diabetes. In the future, effective programs need to be implemented to improve health behaviors to prevent the increasing prevalence of chronic diseases and reduce the burden of disease.

## Figures and Tables

**Table 1.** Characteristics of health interview subjects of the Korea National Health and Nutrition Examination Survey

Characteristics			1998	2001	2005	2007	2008	2009	2010	2011	2012	2013	2014	2015	2016	2017	2018
Total (≥19 yr)			8,991	8,072	7,802	2,983	6,817	7,511	6,352	6,224	5,995	5,792	5,677	5,632	6,129	6,193	6,238
Men			4,180	3,679	3,510	1,242	2,847	3,252	2,745	2,651	2,506	2,485	2,385	2,457	2,647	2,749	2,736
	Age (yr)																
		19-29	886 (21.2)	715 (19.4)	583 (16.6)	141 (11.4)	367 (12.9)	485 (14.9)	308 (11.2)	296 (11.2)	265 (10.6)	320 (12.9)	259 (10.9)	338 (13.8)	308 (11.6)	344 (12.5)	359 (13.1)
		30-39	1,091 (26.1)	944 (25.7)	812 (23.1)	247 (19.9)	600 (21.1)	597 (18.4)	531 (19.3)	459 (17.3)	416 (16.6)	435 (17.5)	412 (17.3)	315 (12.8)	462 (17.5)	409 (14.9)	420 (15.4)
		40-49	888 (21.2)	911 (24.8)	872 (24.8)	248 (20.0)	581 (20.4)	646 (19.9)	542 (19.7)	471 (17.8)	435 (17.4)	506 (20.4)	413 (17.3)	417 (17.0)	510 (19.3)	504 (18.3)	479 (17.5)
		50-59	616 (14.7)	522 (14.2)	579 (16.5)	205 (16.5)	491 (17.2)	545 (16.8)	488 (17.8)	522 (19.7)	474 (18.9)	456 (18.4)	448 (18.8)	506 (20.6)	461 (17.4)	548 (19.9)	506 (18.5)
		60-69	476 (11.4)	381 (10.4)	445 (12.7)	217 (17.5)	441 (15.5)	542 (16.7)	481 (17.5)	471 (17.8)	461 (18.4)	412 (16.6)	434 (18.2)	475 (19.3)	451 (17.0)	475 (17.3)	503 (18.4)
		≥70	223 (5.3)	206 (5.6)	219 (6.2)	184 (14.8)	367 (12.9)	437 (13.4)	395 (14.4)	432 (16.3)	455 (18.2)	356 (14.3)	419 (17.6)	406 (16.5)	455 (17.2)	469 (17.1)	469 (17.1)
	Household income^1^																
		Low	826 (19.8)	582 (16.7)	712 (20.5)	237 (19.9)	554 (20.1)	639 (19.9)	533 (19.6)	531 (20.3)	488 (19.8	500 (20.3)	477 (20.1)	483 (19.8)	534 (20.3)	545 (19.9)	543 (19.9)
		Low-middle	813 (19.4)	718 (20.6)	726 (21.0)	237 (19.9)	552 (20.0)	658 (20.5)	538 (19.8)	514 (19.6)	494 (20.0)	492 (20.0)	476 (20.0)	488 (20.0)	529 (20.1)	543 (19.8)	543 (19.9)
		Middle	864 (20.7)	680 (19.5)	682 (19.7)	238 (19.9)	539 (19.5)	636 (19.8)	580 (21.4)	519 (19.8)	480 (19.5)	492 (20.0)	482 (20.3)	492 (20.2)	519 (19.7)	548 (20.0)	555 (20.4)
		Middle-high	842 (20.1)	695 (20.0)	669 (19.3)	242 (20.3)	572 (20.7)	634 (19.7)	517 (19.1)	521 (19.9)	496 (20.1)	490 (19.9)	475 (20.0)	486 (19.9)	523 (19.8)	550 (20.1)	541 (19.9)
		High	835 (20.0)	804 (23.1)	676 (19.5)	239 (20.0)	543 (19.7)	644 (20.1)	545 (20.1)	534 (20.4)	507 (20.6)	491 (19.9)	467 (19.6)	488 (20.0)	530 (20.1)	554 (20.2)	543 (19.9)
Women			4,811	4,393	4,292	1,741	3,970	4,259	3,607	3,573	3,489	3,307	3,292	3,175	3,482	3,444	3,502
	Age (yr)																
		19-29	1,073 (22.3)	911 (20.7)	753 (17.5)	198 (11.4)	527 (13.3)	565 (13.3)	469 (13.0)	403 (11.3)	399 (11.4)	408 (12.3)	379 (11.5)	355 (11.2)	393 (11.3)	384 (11.1)	405 (11.6)
		30-39	1,122 (23.3)	1,054 (24.0)	930 (21.7)	388 (22.3)	816 (20.6)	833 (19.6)	730 (20.2)	666 (18.6)	601 (17.2)	589 (17.8)	553 (16.8)	454 (14.3)	627 (18.0)	506 (14.7)	496 (14.2)
		40-49	916 (19.0)	961 (21.9)	1,006 (23.4)	317 (18.2)	749 (18.9)	853 (20.0)	641 (17.8)	618 (17.3)	587 (16.8)	631 (19.1)	568 (17.3)	570 (18.0)	633 (18.2)	634 (18.4)	655 (18.7)
		50-59	722 (15.0)	595 (13.5)	650 (15.1)	300 (17.2)	644 (16.2)	707 (16.6)	697 (19.3)	690 (19.3)	663 (19.0)	640 (19.4)	634 (19.3)	658 (20.7)	649 (18.6)	667 (19.4)	692 (19.8)
		60-69	590 (12.3)	497 (11.3)	525 (12.2)	273 (15.7)	640 (16.1)	668 (15.7)	563 (15.6)	577 (16.1)	593 (17.0)	495 (15.0)	540 (16.4)	551 (17.4)	568 (16.3)	626 (18.2)	606 (17.3)
		≥70	388 (8.1)	375 (8.5)	428 (10.0)	265 (15.2)	594 (15.0)	633 (14.9)	507 (14.1)	619 (17.3)	646 (18.5)	544 (16.5)	618 (18.8)	587 (18.5)	612 (17.6)	627 (18.2)	648 (18.5)
	Household income^1^																
		Low	935 (19.4)	753 (18.3)	887 (20.9)	324 (19.6)	776 (20.2)	839 (19.9)	709 (20.0)	715 (20.2)	687 (20.0)	647 (19.7)	665 (20.3)	622 (19.7)	688 (19.8)	685 (20.0)	702 (20.1)
		Low-middle	946 (19.7)	826 (20.0)	823 (19.4)	333 (20.2)	778 (20.2)	852 (20.2)	726 (20.5)	701 (19.8)	699 (20.4)	648 (19.8)	646 (19.8)	634 (20.1)	699 (20.1)	691 (20.2)	702 (20.1)
		Middle	952 (19.8)	784 (19.0)	838 (19.7)	330 (20.0)	758 (19.7)	844 (20.0)	706 (19.9)	701 (19.8)	671 (19.6)	672 (20.5)	646 (19.8)	636 (20.2)	703 (20.3)	683 (19.9)	700 (20.0)
		Middle-high	1,010 (21.0)	836 (20.3)	854 (20.1)	330 (20.0)	771 (20.1)	867 (20.6)	706 (19.9)	705 (19.9)	685 (20.0)	663 (20.2)	663 (20.3)	632 (20.0)	697 (20.1)	686 (20.0)	691 (19.8)
		High	968 (20.1)	922 (22.4)	848 (20.0)	334 (20.2)	759 (19.8)	814 (19.3)	702 (19.8)	712 (20.1)	689 (20.1)	651 (19.8)	649 (19.9)	630 (20.0)	683 (19.7)	683 (19.9)	697 (20.0)

Values are present as number or number (%).

1 Calculated as monthly household income divided by the square root of the number of persons in the household, categorized into quantile by age and gender.

**Table 2.** Trends in the prevalence^1^ of current cigarette smoking^2^

Characteristics			1998	2001	2005	2007	2008	2009	2010	2011	2012	2013	2014	2015	2016	2017	2018	Difference (1998 to 2018)	APC^3^	APC and year of any significant change in trend slope		
																				Before	Change year	After
Total (≥19 yr)			35.1	30.2	28.8	25.3	27.8	27.3	27.5	27.1	25.8	24.1	24.2	22.6	23.9	22.3	22.4	-12.7	-2.1^***^	-2.4^***^	2005	-1.9^***^
Men			66.3	60.9	51.7	45.1	47.8	47.0	48.3	47.3	43.7	42.2	43.2	39.4	40.7	38.1	36.7	-29.6	-2.8^***^	-3.4^***^	2005	-2.4^***^
	Age (yr)																					
		19-29	70.7	64.7	55.5	47.5	53.6	51.9	47.3	44.9	41.5	37.0	34.8	38.7	41.7	37.3	34.9	-35.8	-3.4^***^	-3.1^***^	2008	-3.9^***^
		30-39	71.6	67.9	60.2	58.3	56.4	56.2	60.9	63.7	54.8	54.5	53.2	48.0	51.5	42.7	39.9	-31.7	-2.0^***^	-1.8^***^	2016	-12.9
		40-49	67.9	66.7	55.2	48.8	49.1	48.9	53.6	47.0	49.5	48.0	54.4	45.8	43.9	46.3	44.1	-23.8	-2.3^***^	-3.1^***^	2008	-1.1
		50-59	62.2	55.5	47.7	34.0	41.5	41.6	45.0	44.4	41.8	40.8	39.4	36.5	38.2	37.0	40.6	-21.6	-2.5^***^	-4.0^***^	2007	-1.1^***^
		60-69	58.2	49.9	38.3	31.1	34.5	33.8	30.8	32.5	26.9	32.5	35.8	26.1	25.7	26.6	26.7	-31.5	-4.0^***^	-5.6^***^	2007	-2.3^***^
		≥70	50.5	33.8	27.5	23.3	27.9	23.7	24.7	28.8	23.2	15.6	19.4	17.0	18.0	18.2	14.7	-35.8	-5.3^***^	-7.0	2005	-4.3^***^
	Household income^4^																					
		Low	70.0	68.7	58.8	50.4	55.9	53.4	53.1	54.3	48.7	47.9	46.3	41.8	40.3	42.8	40.1	-29.9	-2.7^***^	-2.4^***^	2011	-3.7^***^
		Low-middle	68.7	61.6	53.9	47.3	49.1	49.9	52.2	50.0	48.0	44.1	42.7	38.3	44.0	43.4	41.8	-26.9	-2.6^***^	-3.4^***^	2005	-2.0^***^
		Middle	65.1	62.4	52.4	46.9	47.6	47.8	45.8	47.0	39.7	43.8	46.4	42.2	39.8	37.3	35.4	-29.7	-2.8^***^	-2.7^***^	2016	-5.8
		Middle-high	65.2	57.1	47.5	45.6	47.5	44.7	46.3	41.7	39.5	38.6	42.7	40.0	43.4	38.5	34.4	-30.8	-2.8^***^	-4.2^***^	2005	-1.9^***^
		High	63.7	54.5	45.5	34.5	38.8	38.4	42.6	42.7	41.2	37.2	37.6	33.9	36.5	28.0	31.0	-32.7	-3.4^***^	-4.5^***^	2007	-2.1^***^
Women			6.5	5.2	5.7	5.3	7.4	7.1	6.3	6.8	7.9	6.2	5.7	5.5	6.4	6.0	7.5	1.0	0.6	1.5	2009	-0.5
	Age (yr)																					
		19-29	5.1	4.4	6.0	7.6	12.7	11.1	7.4	10.4	13.6	9.1	8.9	6.9	7.2	9.7	10.9	5.8	3.4^***^	8.4^***^	2009	-1.9
		30-39	4.5	3.6	4.4	4.4	7.1	7.9	7.6	8.9	9.0	6.9	7.0	6.7	7.6	6.8	8.3	3.8	3.6^***^	6.2^***^	2011	-1.9
		40-49	4.4	3.7	5.7	4.5	5.7	5.5	6.6	4.1	5.5	6.2	5.0	4.9	5.6	5.7	8.7	4.3	2.3^***^	1.2	2016	20.5
		50-59	7.2	4.0	6.7	4.6	3.4	4.1	5.2	5.0	7.9	3.7	2.5	5.4	7.1	3.3	5.0	-2.2	-1.1	-3.5	2008	1.0
		60-69	12.0	6.2	3.5	4.6	4.7	4.5	2.9	3.8	1.6	4.0	2.5	2.8	4.0	2.8	3.6	-8.4	-6.9^***^	-14.3^***^	2005	-2.0
		≥70	14.5	18.0	9.3	6.6	8.7	6.8	5.0	5.1	3.2	3.1	3.8	3.7	3.4	1.9	1.1	-13.4	-10.7^***^	-9.9^***^	2016	-39.1
	Household income^4^																					
		Low	10.2	7.9	8.6	11.3	9.9	11.3	10.6	11.9	11.4	9.3	11.2	9.4	8.9	9.3	10.7	0.5	0.6	1.5	2011	-1.7
		Low-middle	6.7	5.1	6.0	7.3	8.4	7.3	5.7	8.6	10.6	7.8	5.9	5.9	8.2	5.3	10.6	3.9	1.7	2.4	2012	-0.6
		Middle	5.9	3.0	4.8	2.7	7.8	5.9	3.6	4.9	6.5	5.5	4.2	4.7	5.2	7.0	6.9	1.0	1.0	-0.1	2015	11.3
		Middle-high	5.7	3.9	4.3	2.1	4.6	6.6	6.4	3.9	4.2	4.4	4.1	4.2	4.5	4.6	5.6	-0.1	-0.4	-2.9	2005	0.9
		High	4.9	5.7	4.9	2.7	6.5	3.8	4.9	3.9	5.8	4.3	2.4	3.6	5.3	3.4	3.2	-1.7	-1.7	0.0	2008	-3.6

Values are presented as weighted %.

1 Age-standardized prevalence was calculated using the 2005 population projections for Korea.

2 Current cigarette smoking: percentage of adults who have smoked at least 100 cigarettes during their lifetime and who are currently smokers.

3 The annual percent change (APC) is significantly different from 0.

4 Calculated as monthly household income divided by the square root of the number of persons in the household, categorized into quantile by age and gender.

*** p<0.001.

**Table 3.** Trends in the prevalence^1^ of exposure to secondhand smoking

Exposures		2005	2007	2008	2009	2010	2011	2012	2013	2014	2015	2016	2017	2018	Difference (2005 to 2018)	APC^2^	APC and year of any significant change in trend slope		
																	Before	Change year	After
At home (≥19 yr)^3^																			
	Total	18.5	14.7	15.5	14.9	14.9	12.5	11.9	10.9	10.7	8.2	6.4	4.7	4.0	-14.5	-8.8^***^	-5.9^***^	2014	-22.7^***^
	Men	7.1	4.4	6.5	6.9	5.8	4.9	4.8	5.5	4.8	4.2	4.0	2.3	1.0	-6.1	-7.3^***^	-4.6^***^	2016	-50.1^***^
	Women	24.1	20.5	20.5	19.4	19.8	16.7	16.0	14.1	13.9	10.7	7.9	6.3	6.1	-18.0	-8.7^***^	-6.1^***^	2014	-21.0^***^
Workplace (≥19 yr)^4^																			
	Total	36.9	46.0	45.4	45.8	49.2	45.2	46.1	47.4	40.1	26.9	17.4	12.7	11.5	-25.4	-5.3^***^	2.7^***^	2013	-26.9^***^
	Men	44.6	55.2	53.4	53.3	58.7	55.3	54.5	57.3	49.1	36.2	23.5	17.3	14.4	-30.2	-3.9	2.9^***^	2013	-25.1^***^
	Women	31.8	36.4	38.2	39.5	41.8	37.2	38.9	38.7	32.8	18.5	12.1	8.5	8.7	-23.1	-5.4^***^	2.3	2013	-29.9^***^
Public places (≥19 yr)^5^																			
	Total	-	-	-	-	-	-	-	58.0	52.2	35.4	22.3	21.1	16.9	-41.1	-23.2^***^	-24.7^***^	2016	-19.4
	Men	-	-	-	-	-	-	-	62.6	56.3	41.0	26.9	26.0	19.1	-43.5	-21.3^***^	-19.3^***^	2015	-23.2^***^
	Women	-	-	-	-	-	-	-	55.4	49.8	31.8	19.4	17.9	15.4	-40.0	-24.6^***^	-26.0^***^	2016	-20.8

Values are presented as weighted %.

1 Age-standardized prevalence was calculated using the 2005 population projections for Korea.

2 The annual percent change (APC) is significantly different from 0.

3 Secondhand smoking at home: percentage of current non-smokers who have been exposed to secondhand smoke at home during the past 7 days.

4 Secondhand smoking in the workplace: percentage of current non-smokers who have been exposed to secondhand smoke in the workplace during the past 7 days.

5 Secondhand smoking in public places: percentage of current non-smokers who have been exposed to secondhand smoke in public places during the past 7 days.

*** p<0.001.

**Table 4.** Trends in the prevalence^1^ of current alcohol drinking^2^

Characteristics			2005	2007	2008	2009	2010	2011	2012	2013	2014	2015	2016	2017	2018	Difference (2005 to 2018)	APC^3^	APC and year of any significant change in trend slope		
																		Before	Change year	After
Total (≥19 yr)			54.6	57.3	59.6	59.4	60.5	60.7	57.9	60.2	60.0	60.6	61.9	62.1	60.6	6.0	0.7^***^	2.8^***^	2008	0.3
Men			72.6	73.5	74.7	75.8	77.8	77.7	73.6	75.3	74.4	75.2	75.3	74.0	70.5	-2.1	-0.1	1.3^***^	2010	-0.8^***^
	Age (yr)																			
		19-29	78.4	74.6	75.6	82.7	81.6	84.8	74.9	81.2	77.1	76.9	76.8	74.2	63.5	-14.9	-0.7	1.2	2011	-2.3^***^
		30-39	77.8	79.9	79.9	78.1	84.9	81.9	79.0	78.5	79.0	80.0	82.6	78.6	75.2	-2.6	-0.1	1.3	2010	-0.8
		40-49	76.1	79.4	80.5	79.1	79.8	75.4	73.8	78.7	77.8	79.3	76.5	77.6	77.7	1.6	0.0	1.4	2008	-0.3
		50-59	71.5	75.8	74.1	75.1	78.1	81.2	75.6	74.9	72.1	74.0	74.1	73.4	71.5	0.0	-0.2	1.5^***^	2011	-1.4^***^
		60-69	60.7	58.3	65.6	66.4	66.6	70.6	64.8	67.4	65.0	65.0	68.2	69.9	69.8	9.1	0.8^***^	2.4	2009	0.4
		≥70	45.3	47.8	50.3	50.5	51.1	51.4	58.8	47.6	57.6	57.8	55.8	53.2	53.6	8.3	1.1^***^	2.2^***^	2015	-2.7
	Household income^4^																			
		Low	66.2	71.1	70.5	70.3	74.8	73.1	74.6	71.9	68.6	68.4	67.9	66.6	66.9	0.7	-0.2	1.8^***^	2011	-1.8^***^
		Low-middle	71.3	68.6	74.7	75.8	75.7	75.0	72.2	71.6	72.5	70.8	74.2	74.0	70.8	-0.5	-0.1	1.4	2009	-0.5
		Middle	74.8	72.0	77.5	75.0	79.1	80.6	72.9	75.9	69.8	75.6	75.8	77.6	67.8	-7.0	-0.2	0.8	2011	-1.0
		Middle-high	74.8	75.9	75.1	78.0	80.4	80.6	71.4	78.3	78.8	80.3	78.7	72.5	70.4	-4.4	-0.1	0.4	2016	-6.5
		High	76.4	80.0	77.5	80.7	80.1	80.1	77.0	79.3	82.1	81.0	80.3	79.2	77.5	1.1	0.2	0.5^***^	2015	-1.5
Women			37.0	41.5	45.0	43.4	43.3	44.2	42.9	45.7	46.4	46.5	48.9	50.5	51.2	14.2	2.0^***^	5.0^***^	2008	1.6^***^
	Age (yr)																			
		19-29	51.8	63.1	63.8	55.4	52.1	60.2	57.7	62.3	60.3	56.6	64.1	66.3	65.7	13.9	1.3^***^	3.8	2008	0.8
		30-39	41.2	43.5	50.3	50.1	49.1	52.6	48.0	56.4	53.9	50.6	55.5	58.6	60.0	18.8	2.3^***^	6.1	2008	1.8^***^
		40-49	38.6	40.6	47.5	48.6	50.6	43.3	44.6	43.1	50.4	52.9	52.3	54.7	55.1	16.5	2.2^***^	6.1	2008	1.7^***^
		50-59	28.6	36.0	34.4	33.9	36.4	34.9	35.7	39.1	36.6	42.1	42.0	39.0	41.4	12.8	2.4^***^	5.8	2008	2.0^***^
		60-69	17.9	20.6	20.4	23.2	23.4	24.8	23.2	20.3	21.1	29.3	26.0	27.1	30.2	12.3	3.3^***^	2.3	2014	5.4
		≥70	14.5	10.4	15.0	13.8	15.5	14.4	16.3	15.3	20.3	13.8	13.5	16.6	12.5	-2.0	0.2	2.8	2014	-5.7
	Household income^4^																			
		Low	36.6	40.2	44.6	41.7	44.0	44.2	41.6	40.8	44.0	44.6	46.6	50.4	49.6	13.0	1.9^***^	1.4^***^	2015	4.2
		Low-middle	37.9	43.7	46.4	42.0	42.4	41.9	42.9	42.6	47.3	45.1	45.7	49.8	56.4	18.5	2.2^***^	1.2^***^	2016	10.8
		Middle	38.1	42.0	46.3	44.0	43.0	48.6	44.5	46.4	48.2	42.4	47.6	55.6	48.8	10.7	1.7^***^	5.0	2008	1.4
		Middle-high	38.6	44.1	41.6	45.4	47.0	45.3	43.6	45.7	46.6	48.1	52.6	48.7	49.4	10.8	1.8^***^	3.6	2009	1.3^***^
		High	34.1	38.3	46.3	43.7	39.6	42.5	43.5	53.3	45.8	52.0	50.8	48.1	51.6	17.5	2.7^***^	8.5	2008	1.8^***^

Values are presented as weighted %.

1 Age-standardized prevalence was calculated using the 2005 population projections for Korea.

2 Current alcohol drinking: percentage of adults who have had alcoholic drinks 1 or more times a month during the past year.

3 The annual percent change (APC) is significantly different from 0.

4 Calculated as monthly household income divided by the square root of the number of persons in the household, categorized into quantile by age and gender.

*** p<0.001.

**Table 5.** Trends in the prevalence^1^ of binge drinking^2^

Characteristics			2005	2007	2008	2009	2010	2011	2012	2013	2014	2015	2016	2017	2018	Difference (2005 to 2018)	APC^3^	APC and year of any significant change in trend slope		
																		Before	Change year	After
Total (≥19 yr)			36.2	37.1	39.5	40.0	39.9	39.0	38.0	37.4	37.5	38.7	39.3	39.0	38.9	2.7	0.2	2.9	2008	-0.2
Men			55.3	53.7	56.7	57.6	57.6	55.9	53.4	53.2	53.1	54.2	53.5	52.7	50.8	-4.5	-0.7^***^	1.0	2009	-1.2^***^
	Age (yr)																			
		19-29	60.6	59.2	60.6	62.6	63.3	63.2	55.3	56.2	56.8	58.7	53.5	54.8	46.8	-13.8	-1.4^***^	0.9	2010	-2.7^***^
		30-39	62.1	62.1	63.1	62.2	66.8	65.1	60.5	59.4	60.8	64.1	62.8	57.9	55.9	-6.2	-0.6	-0.1	2016	-5.8
		40-49	61.9	61.9	62.7	64.1	61.4	54.6	55.3	59.3	56.2	56.5	58.5	59.1	59.3	-2.6	-0.6^***^	-1.0^***^	2015	1.6
		50-59	52.3	49.1	60.4	58.5	57.5	59.0	57.9	54.5	52.4	51.3	53.0	52.5	51.5	-0.8	-0.7	5.0	2008	-1.5^***^
		60-69	38.2	35.4	39.6	45.2	38.1	42.0	40.2	41.1	39.7	42.0	42.3	43.3	47.0	8.8	1.0^***^	0.4	2016	5.5
		≥70	25.0	17.8	20.7	22.3	24.6	19.4	27.4	19.0	26.4	22.2	23.9	23.0	23.9	-1.1	0.5	-2.8	2008	0.9
	Household income^4^																			
		Low	51.7	57.0	55.5	54.0	55.0	50.9	55.1	49.8	51.5	49.0	47.2	48.3	47.8	-3.9	-1.0^***^	2.4	2008	-1.7^***^
		Low-middle	51.9	51.6	56.7	60.7	56.0	54.0	52.6	48.7	52.5	50.5	54.8	55.8	52.6	0.7	-0.3	2.6	2009	-1.1
		Middle	55.6	53.1	58.9	57.2	59.3	55.5	55.0	55.9	48.0	56.2	51.9	53.7	47.7	-7.9	-0.8^***^	0.8	2010	-1.8^***^
		Middle-high	59.6	53.7	58.5	58.3	59.9	58.7	46.6	56.3	54.6	59.1	57.2	51.7	51.5	-8.1	-0.8^***^	-0.5	2016	-4.4
		High	57.7	54.8	55.7	58.1	59.4	61.6	56.9	56.1	58.4	57.5	56.5	54.1	55.3	-2.4	-0.3	0.8	2011	-1.1^***^
Women			17.2	20.5	22.3	22.2	22.1	22.1	22.9	21.9	22.5	23.3	25.0	25.0	26.9	9.7	2.4^***^	7.5^***^	2008	1.7^***^
	Age (yr)																			
		19-29	30.7	41.9	39.0	36.2	35.2	38.9	35.9	37.2	39.5	37.0	45.7	45.9	49.8	19.1	2.8^***^	1.7	2015	8.2
		30-39	17.9	18.0	25.9	23.9	25.9	23.7	28.0	26.5	24.2	25.1	27.1	26.0	27.1	9.2	2.4^***^	12.1^***^	2008	0.9
		40-49	17.8	17.7	23.1	23.8	22.2	21.0	21.4	18.5	21.8	23.6	23.0	22.8	26.0	8.2	1.5^***^	8.0	2008	0.6
		50-59	10.7	15.2	10.9	14.7	16.3	15.9	17.9	16.5	15.3	17.3	16.5	16.2	17.9	7.2	3.0^***^	7.3^***^	2011	0.3
		60-69	3.9	5.5	4.8	7.9	5.4	6.6	7.1	6.2	6.1	10.0	7.4	9.6	8.5	4.6	5.5^***^	12.8	2009	4.0^***^
		≥70	1.8	1.0	2.0	1.4	2.2	1.6	1.8	2.0	2.7	3.4	1.0	2.0	1.0	-0.8	2.2	7.1^***^	2015	-24.5
	Household income^4^																			
		Low	17.4	23.9	27.9	24.7	25.8	25.5	25.0	20.9	23.4	26.6	27.4	29.8	29.3	11.9	2.4^***^	12.1	2008	1.1
		Low-middle	19.6	21.5	23.6	22.1	20.5	21.4	23.6	23.0	23.7	23.2	24.7	25.6	29.9	10.3	2.5^***^	1.6^***^	2016	10.5
		Middle	17.1	19.7	22.2	21.5	22.4	24.0	23.6	22.1	20.0	24.0	26.9	27.0	26.2	9.1	2.7^***^	7.7	2008	2.1^***^
		Middle-high	18.0	21.9	17.8	23.8	22.5	20.6	22.6	20.7	22.6	20.5	24.2	21.3	24.9	6.9	1.4^***^	5.0	2009	0.5
		High	14.3	14.3	20.2	18.2	18.2	19.3	19.6	23.0	22.8	22.4	20.9	21.1	24.1	9.8	3.4^***^	9.9	2008	2.3^***^

Values are presented as weighted %.

1 Age-standardized prevalence was calculated using the 2005 population projections for Korea.

2 Binge drinking: percentage of adults who have drunk ≥ 7 (men) or ≥ 5 (women) alcoholic drinks more than once a week during the past year.

3 The annual percent change (APC) is significantly different from 0.

4 Calculated as monthly household income divided by the square root of the number of persons in the household, categorized into quantile by age and gender.

*** p<0.001.

**Table 6.** Trends in the prevalence^1^ of aerobic physical activity (PA)^2^

Characteristics			2007	2008	2009	2010	2011	2012	2013	2014	2015	2016	2017	2018	Difference (2007 to 2018)	APC^3^	APC and year of any significant change in trend slope		
																	Before	Change year	After
Total (≥19 yr)			72.5	75.5	76.4	71.8	67.8	68.1	70.3	58.3	52.7	49.4	48.5	47.6	-24.9	-4.5^***^	-2.5^***^	2013	-7.9^***^
Men			76.3	79.2	80.4	75.7	72.1	73.1	76.8	62.0	55.8	52.5	50.6	51.0	-25.3	-4.2^***^	-1.9^***^	2013	-8.3^***^
	Age (yr)																		
		19-29	83.6	85.8	87.6	81.9	77.2	79.7	85.9	79.8	72.1	66.9	67.0	69.7	-13.9	-2.2^***^	-0.8	2013	-4.6^***^
		30-39	72.9	75.5	75.2	74.6	69.0	69.9	74.0	58.2	54.3	50.7	51.3	55.3	-17.6	-3.6^***^	-1.9^***^	2013	-6.7^***^
		40-49	73.5	75.0	79.4	74.4	70.7	75.1	76.4	57.5	55.1	50.7	48.4	48.8	-24.7	-4.2^***^	-1.1	2013	-9.3^***^
		50-59	80.4	80.7	82.4	74.5	72.6	71.8	73.0	60.2	48.3	44.7	45.9	36.5	-43.9	-6.1^***^	-3.0^***^	2013	-12.1^***^
		60-69	79.1	83.1	81.4	76.3	72.0	71.3	73.2	53.7	46.6	50.9	34.4	40.8	-38.3	-6.4^***^	-3.5^***^	2013	-12.0^***^
		≥70	61.4	75.0	71.9	64.8	69.7	61.5	70.6	48.2	38.3	35.6	34.8	27.7	-33.7	-6.6^***^	-1.5	2013	-17.1^***^
	Household income^4^																		
		Low	72.3	79.4	77.8	72.6	72.2	71.1	74.1	59.9	55.6	43.1	49.7	48.4	-23.9	-4.6^***^	-2.1	2013	-8.6^***^
		Low-middle	70.0	78.7	78.2	75.2	71.4	66.6	70.6	60.4	56.5	52.7	48.9	48.6	-21.4	-4.5^***^	3.9	2009	-5.4^***^
		Middle	79.3	76.5	82.5	76.6	70.2	72.5	77.1	62.7	52.5	51.7	51.1	46.6	-32.7	-4.4^***^	-1.7	2013	-9.4^***^
		Middle-high	77.4	80.4	81.2	74.2	73.7	78.3	77.1	65.4	52.7	54.5	51.2	54.6	-22.8	-4.0^***^	-1.7	2013	-7.9^***^
		High	82.2	82.2	82.2	80.8	73.8	78.0	85.1	61.5	61.5	60.4	51.9	57.6	-24.6	-3.4^***^	-0.8	2013	-7.9^***^
Women			68.9	72.1	72.5	68.0	63.8	63.3	64.0	54.7	49.8	46.4	46.6	44.0	-24.9	-4.7^***^	2.5	2009	-5.5^***^
	Age (yr)																		
		19-29	69.7	74.2	75.7	69.4	68.2	72.5	69.2	64.1	60.8	56.4	63.9	57.1	-12.6	-2.4^***^	2.4	2009	-2.8^***^
		30-39	64.2	70.1	68.0	68.7	61.8	63.9	61.1	57.8	48.3	47.4	43.6	45.8	-18.4	-4.2^***^	0.9	2010	-5.6^***^
		40-49	75.5	75.0	74.8	69.4	65.8	62.8	68.6	56.9	54.0	45.0	46.6	42.8	-32.7	-5.0^***^	-3.1^***^	2013	-8.4^***^
		50-59	73.3	76.8	77.2	71.0	67.1	61.3	66.1	52.6	44.2	46.0	43.0	39.2	-34.1	-6.2^***^	-4.2^***^	2013	-9.6^***^
		60-69	68.0	68.9	72.9	68.7	63.2	59.6	59.8	42.7	44.9	41.0	37.1	36.6	-31.4	-6.4^***^	5.0	2009	-7.7^***^
		≥70	53.8	58.9	59.9	50.7	44.6	43.8	45.2	29.1	24.2	23.9	22.0	20.5	-33.3	-10.1^***^	4.5	2009	-11.7^***^
	Household income^4^																		
		Low	68.6	67.9	72.9	70.1	60.4	61.6	58.1	51.9	48.7	42.5	45.1	40.8	-27.8	-5.4^***^	4.7	2009	-6.3^***^
		Low-middle	65.6	71.5	72.7	67.2	65.1	62.1	65.1	56.9	48.3	41.6	45.6	43.3	-22.3	-4.8^***^	-2.5	2013	-8.6^***^
		Middle	72.1	76.5	72.7	68.8	62.4	65.4	65.0	52.1	50.0	45.8	44.7	44.2	-27.9	-5.3^***^	-3.9^***^	2013	-7.8^***^
		Middle-high	69.7	74.2	73.2	67.0	66.0	61.2	64.3	55.3	47.6	52.8	46.2	45.4	-24.3	-4.6^***^	0.7	2009	-5.3^***^
		High	69.2	71.5	70.9	66.8	65.5	66.8	68.0	56.9	53.7	49.4	51.3	46.7	-22.5	-3.7^***^	-1.6^***^	2013	-6.9^***^

Values are presented as weighted %.

1 Age-standardized prevalence was calculated using the 2005 population projections for Korea.

2 Aerobic PA: percentage of adults who have performed 150 minutes of moderate-intensity PA or 75 minutes of vigorous-intensity PA or an equivalent combination of moderate- and vigorous-intensity PA in a typical week.

3 The annual percent change (APC) is significantly different from 0.

4 Calculated as monthly household income divided by the square root of the number of persons in the household, categorized into quantile by age and gender.

*** p<0.001.

**Table 7.** Trends in the prevalence^1^ of healthy behaviors practice^2^

Characteristics			2007	2008	2009	2010	2011	2012	2013	2014	2015	2016	2017	2018	Difference (2007 to 2018)	APC^3^	APC and year of any significant change in trend slope		
																	Before	Change year	After
Total (≥19 yr)			25.1	25.1	25.1	23.7	21.4	23.8	22.6	19.2	17.4	15.2	14.8	14.5	-10.6	-5.3^***^	-2.6^***^	2013	-9.5^***^
Men			12.5	13.1	12.3	11.1	10.2	14.4	13.1	10.9	10.5	9.4	8.8	10.7	-1.8	-2.5^***^	-0.4	2013	-5.3
	Age (yr)																		
		19-29	13.5	13.5	10.9	11.7	7.6	17.9	10.3	12.3	13.3	11.4	12.1	17.4	3.9	1.5	-0.6	2016	17.1
		30-39	6.0	8.7	8.2	5.3	5.0	10.9	12.0	9.7	8.7	6.6	7.4	10.0	4.0	1.7	6.3	2013	-4.0
		40-49	9.2	9.0	9.0	11.0	11.6	10.8	10.5	8.0	6.9	9.2	6.6	8.1	-1.1	-2.5	7.5	2011	-6.3^***^
		50-59	14.5	15.3	15.6	9.4	9.1	12.2	12.6	12.3	10.7	8.8	8.1	7.2	-7.3	-5.8^***^	-4.2	2015	-12.7
		60-69	24.1	20.3	17.5	16.9	16.1	22.0	16.4	11.2	13.2	10.9	7.1	9.2	-14.9	-7.7^***^	-2.1	2012	-12.5^***^
		≥70	20.9	24.8	27.0	24.0	24.9	19.9	29.6	16.3	14.7	12.6	13.0	8.6	-12.3	-8.1^***^	-0.2	2013	-17.9^***^
	Household income^4^																		
		Low	12.8	13.4	13.4	13.1	10.3	12.0	13.9	11.9	12.0	12.0	9.9	9.4	-3.4	-2.4^***^	-1.3	2016	-11.8
		Low-midd	le 15.5	11.8	10.8	11.5	12.4	12.3	14.0	9.7	14.3	8.5	8.5	9.2	-6.3	-2.8^***^	-0.8	2015	-10.4
		Middle	13.7	11.6	13.7	10.5	9.1	16.3	13.1	12.1	10.0	8.7	8.3	11.2	-2.5	-2.5	0.3	2013	-6.3
		Middle-high	10.9	14.6	13.0	9.9	7.8	14.9	12.3	12.2	8.2	8.4	9.7	14.2	3.3	-1.1	-3.9	2016	19.8
		High	8.3	13.4	10.2	10.6	11.0	16.6	11.8	8.8	7.8	9.3	7.2	9.3	1.0	-3.1	5.3	2012	-9.1^***^
Women			37.3	36.8	37.8	36.2	32.5	32.9	31.9	27.1	24.2	21.0	20.9	18.3	-19.0	-6.4^***^	-3.5^***^	2013	-10.7^***^
	Age (yr)																		
		19-29	21.2	24.7	30.5	30.1	23.1	25.6	22.9	23.9	23.6	19.6	18.9	15.9	-5.3	-3.8^***^	20.7	2009	-5.8^***^
		30-39	35.4	30.9	29.8	34.4	25.1	32.4	24.4	25.3	23.3	18.8	18.4	16.1	-19.3	-5.9^***^	-2.2	2012	-9.3^***^
		40-49	41.7	38.4	36.8	32.8	37.7	32.2	34.4	28.1	24.2	18.0	21.6	17.9	-23.8	-6.8^***^	-3.5	2013	-12.0^***^
		50-59	46.6	48.8	48.3	43.5	41.6	36.5	41.9	31.2	24.3	25.4	25.6	21.6	-25.0	-7.3^***^	-4.7^***^	2013	-11.2^***^
		60-69	49.5	51.8	53.5	49.6	45.9	44.5	46.5	33.0	30.9	31.1	25.7	26.3	-23.2	-6.8^***^	-3.9^***^	2013	-11.1^***^
		≥70	46.2	45.3	47.2	38.9	34.2	36.1	36.5	24.1	19.8	19.2	18.0	16.1	-30.1	-9.7^***^	-6.6^***^	2013	-14.7^***^
	Household income^4^																		
		Low	33.4	34.3	38.6	35.5	29.3	31.1	32.0	26.4	24.5	19.9	19.9	18.4	-15.0	-6.1^***^	8.1	2009	-7.5^***^
		Low-middle	33.6	35.8	38.1	37.0	34.7	32.7	35.6	27.5	24.9	20.4	20.6	14.0	-19.6	-6.6^***^	-1.1	2013	-15.0^***^
		Middle	43.4	37.1	37.6	37.3	30.5	32.4	29.8	24.9	25.6	19.1	17.7	21.7	-21.7	-6.9^***^	-4.7	2010	-7.5^***^
		Middle-high	36.0	40.5	35.3	33.5	32.7	32.3	32.6	26.9	23.3	22.3	21.8	17.2	-18.8	-6.2^***^	-3.7^***^	2013	-10.4^***^
		High	40.7	36.3	38.7	37.4	34.5	35.7	29.5	29.5	22.6	23.4	25.1	20.3	-20.4	-5.7^***^	-3.2	2012	-8.2^***^

Values are presented as weighted %.

1 Age-standardized prevalence was calculated using the 2005 population projections for Korea.

2 Healthy behaviors practice: percentage of adults who met the guidelines for cigarette smoking, alcohol drinking, and physical activity.

3 The annual percent change (APC) is significantly different from 0.

4 Calculated as monthly household income divided by the square root of the number of persons in the household, categorized into quantile by age and gender.

*** p<0.001.

## SUPPLEMENTARY MATERIALS

Supplementary Material 1. Trends in health behaviors in Korea National Health and Nutrition Examination Survey.

Korean version is available at http://www.e-epih.org/.
