# Supplementary material for: Trends in health behaviors over 20 years: findings from the 1998-2018 Korea National Health and Nutrition Examination Survey
Source: Epidemiol Health. 2021 Apr 19;43:e2021026. doi: 10.4178/epih.e2021026 (PMC8289472; doi:10.4178/epih.e2021026)
Supplement: Supplementary Material 1. — Trends in health behaviors in Korea National Health and Nutrition Examination Survey. [file epih-43-e2021026-suppl1.docx]

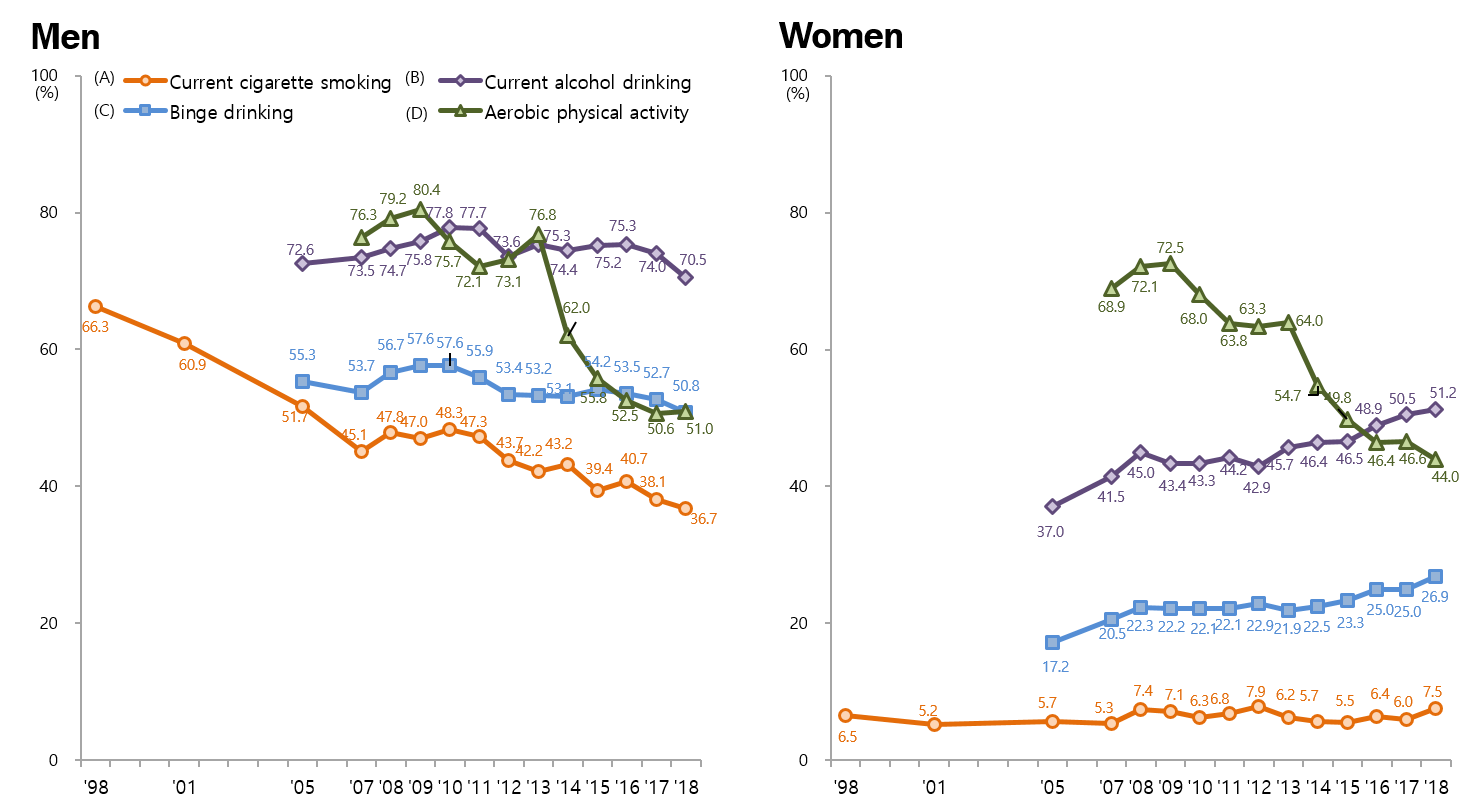


**Supplementary Material 1. Trends in health behaviors in Korea National Health and Nutrition Examination Survey.** (A) Current cigarette smoking: percentage of adults who have smoked at least 100 cigarettes during their lifetime and who are currently smokers, (B) Current alcohol drinking: percentage of adults who have had alcoholic drinks 1 or more times a month during the past year, (C) Binge drinking: percentage of adults who have drunk ≥ 7 (men) or ≥ 5 (women) alcoholic drinks more than once a week during the past year, (D) Aerobic physical activity: percentage of adults who have performed 150 minutes of moderate-intensity physical activity or 75 minutes of vigorous-intensity physical activity or an equivalent combination of moderate- and vigorous-intensity physical activity in a typical week.
