# Supplementary material for: Trends in health behaviors over 20 years: findings from the 1998-2018 Korea National Health and Nutrition Examination Survey
Source: Epidemiol Health. 2021 Apr 19;43:e2021026. doi: 10.4178/epih.e2021026 (PMC8289472; doi:10.4178/epih.e2021026)
Supplement: Supplementary file 2 [file epih-43-e2021026-suppl2.docx]

**Title:**

(국문) 국민건강영양조사 20년(1998-2018): 건강행태 추이

(영문) Trends in health behaviors over 20 years: findings from the 1998-2018 Korea National Health and Nutrition Examination Survey

**Running title**: Twenty-year trends in health behavior

**Authors:**

김소연^1^, 최선혜^1^, 김지희^1^, 박수연^1^, 김영택^2^, 박옥^1^, 오경원^1^

Soyeon Kim^1^, Sunhye Choi^1^, Jihee Kim^1^, Suyeon Park^1^, Youngtaek Kim^2^, Ok Park^1^, Kyungwon Oh^1^

**Affiliations:**

^1^질병관리청 만성질환관리국 건강영양조사분석과, ^2^충남대학교 병원

^1^Division of Health and Nutrition Survey and Analysis, Bureau of Chronic Disease Prevention and Control, Korea Disease Control and Prevention Agency, Cheongju, Korea; ^2^Public Health Medical Service Office, Chungnam National University Hospital, Daejeon, Korea

**Correspondence:** Kyungwon Oh

Division of Health and Nutrition Survey and Analysis, Bureau of Chronic Disease Prevention and Control, Korea Disease Control and Prevention Agency, 187 Osongsaengmyeong 2-ro, Heungduk-gu, Cheongju 28159, Korea

E-mail: [kwoh27@korea.kr](mailto:kwoh27@korea.kr)

**ABSTRACT**

**목적**: 국민건강영양조사 자료를 이용하여 우리나라 성인의 건강행태 현황 및 추이를 파악하였다.

**방법**: 조사대상은 국민건강영양조사 제1기(1998)~제7기(2016-2018) 건강설문조사(흡연, 음주, 신체활동)에 참여한 만19세이상 96,408명이었다. 건강행태 분율과 연간변화율(Annual percent change)은 SAS 및 Joint point 프로그램을 사용하여 산출하였다.

**결과**: 남자 현재흡연율은 20년간 매년 2.8%p 정도 감소하였고(APC=-2.8, p<0.001), 가정 내 간접흡연노출률은 2005년에 비해 크게 감소하였다(APC=-8.8, p<0.001). 월간음주율은 남자에서 변화가 없었던 반면, 여자에서는 2005년에 비해 증가하였으며(APC=2.0, p<0.001), 월간폭음률은 남자에서 2005년 대비 감소한 반면(APC=-0.7, p<0.001), 여자에서는 증가하였다(APC=2.4, p<0.001). 유산소신체활동 실천율은 2014년 이후 남녀 모두에서 감소하였다(p<0.001). 건강생활실천율(비흡연, 금주, 유산소신체활동실천) 역시 감소 경향이었으며(APC=-5.3, p<0.001), 특히 여자에서 크게 감소하였다(APC=-6.4, p<0.001).

**결론**: 지난 20년간 흡연행태(흡연, 간접흡연노출)는 개선된 반면, 음주행태는 큰 변화가 없었고, 신체활동 지표(신체활동 실천)는 크게 악화되었다. 건강행태는 만성질환 발생과 관련된 주요한 요인이므로 건강행태 개선을 위한 보다 적극적인 프로그램 추진이 필요하다.

**주제어**: 국민건강영양조사, 건강행태, 흡연, 음주, 신체활동

**INTRODUCTION**

비전염성질환(Noncommunicable disease, NCD, 만성질환)으로 인한 사망은 전세계 사망의 약 65%를 차지하고 있다 [1]. 우리나라도 암, 당뇨병, 심뇌혈관질환 등 만성질환에 의한 사망이 전체 사망의 80% 이상을 차지하고 있다 [2].

흡연, 음주, 신체활동 등 건강행태는 고혈압, 이상지질혈증과 같은 만성질환의 선행질환 [3], 암, 심뇌혈관질환 등을 포함한 만성질환의 발생, 이로 인한 질병부담 및 조기 사망과 관련이 있다 [4-5]. 또한, 금연, 절주, 규칙적인 신체활동은 독립적으로 또는 복합적으로 건강관련 삶의 질, 건강 수명과도 밀접하게 관련되어 있다 [6-10]. 이러한 이유로 우리나라를 비롯한 대부분의 국가에서 건강행태 교정을 통한 건강증진 및 만성질환 예방을 건강정책의 우선 순위로 설정하여 추진하고 있다. 우리나라는 국민건강증진종합계획 2020에서 건강생활 실천 확산을 목표로 금연, 절주, 신체활동, 영양 분야의 주요 지표의 추이, 소득수준 등 건강형평성에 따른 지표별 격차에 관한 목표치를 설정하고 국민건강영양조사를 통해 건강증진 프로그램의 성과를 모니터링하고 있다.

이 연구에서는 국민건강영양조사(1998년~2018년) 자료를 이용하여 흡연, 음주, 신체활동 등 주요 건강행태의 변화를 파악하여 만성질환 예방 및 관리에 필요한 근거 자료를 제공하고자 하였다.

**SUBJECTS AND METHODS**

**조사대상**

국민건강영양조사는 우리 국민의 건강 및 영양 상태에 관한 국가 단위의 통계를 산출하기 위한 조사로 검진조사, 건강설문조사, 영양조사로 구성되어 있다. 조사표본은 1998년부터 조사구 및 가구를 1, 2차 추출단위로 한 2단계 층화집락표본추출방법을 동일하게 적용하여 추출하였다. 기본 추출틀은 인구주택총조사자료이며(2010-2012년은 예외적으로 설계시점에서 인구주택총조사자료가 노후화되어 주민등록인구 및 아파트단지시세조사자료를 사용), 층화변수는 시도, 동읍면, 주택유형(일반/아파트)을 적용하였다. 2010년이후부터는 표본설계 시 내재적 층화변수로 주거면적, 가구주 학력 등을 추가로 고려하여 변화된 모집단 특성을 반영함과 동시에 표본의 대표성과 시계열적 일관성을 유지한 통계 생산이 가능하도록 하였다.

조사표본은 위의 방법을 이용하여 추출된 연간 약 200개 조사구, 조사구당 20~23개 가구였고, 조사대상은 표본가구 내 만1세 이상의 모든 가구원, 약 10,000명이었다. 본 연구의 분석 대상은 국민건강영양조사 제1기(1998)부터 제7기(2016-2018) 건강설문조사에 참여한 만19세 이상이었다.

**건강설문조사**

건강설문조사는 면접조사(만성질환 의사진단 및 관리, 병원이용, 손상 등)와 자기기입조사(흡연, 음주 등)로 구분되고, 연령에 따라 소아(만1-11세), 청소년(만12-18세), 성인(만19세이상) 조사표의 문항이 약간 차이가 있도록 설계되었다. 본 연구에서는 건강설문조사 중 자기기입식 방법(신체활동의 경우 2014년부터 면접조사로 변경)에 의해 수집한 흡연, 음주, 신체활동 조사자료에 국한하여 분석하였다.

흡연 영역의 경우 평생 및 현재 흡연, 가정, 직장, 공공장소실내에서의 간접흡연에 대해 조사하였다. 가정과 직장실내 간접흡연은 2013년부터 최근 7일이내의 경험에 대해서만 응답하도록 준거 기간을 추가하였고, 공공장소 간접흡연 문항은 2013년 도입 시부터 준거기간을 추가하였다. 음주는 2005년부터 알코올사용장애 선별검사(alcohol use disorder identification test, AUDIT)를 도입하여 음주 빈도 및 음주량, 폭음 빈도 등에 대해 조사하였다. 신체활동 영역에서는 2005년부터 국제신체활동설문(international physical activity questionnaires, IPAQ short form)을 도입하였다. 이 설문 도구는 고강도 신체활동 일수와 시간, 중강도 신체활동 일수와 시간, 걷기 일수와 시간, 앉아서 보내는 시간의 총 7문항으로 구성되어 있다. 2005년에는 신체활동 시간을 범주형으로 질문하여 정확한 시간을 확인하는 데에 제한적이어서 2007년부터는 IPAQ 원문과 동일하게 신체활동 시간을 개방형으로 응답하도록 변경하였다. 2014년부터는 영역별(일, 여가, 이동) 신체활동량을 파악할 수 있도록 GPAQ(global physical activity questionnaires)을 도입하였다. GPAQ은 일, 이동, 여가활동을 구분하여 각각에 해당하는 중강도, 고강도 활동 시간, 앉아서 보내는 시간 등 총 16문항으로 구성되어 있으며, 문항의 난이도를 고려하여 면접조사로 실시하였다. 근력 운동은 2005년에 도입한 이후 자구 수정 이외에는 큰 변화 없이 조사하였다.

**건강행태 지표 정의**

현재흡연자는 평생 담배 5갑을 피웠고 현재 담배를 피우는 사람으로 정의하였고, 가정실내/공공장소 간접흡연 경험률은 현재 비흡연자(과거흡연자 포함)를 분모로 가정실내/공공장소에서 다른 사람이 피우는 담배 연기를 맡은 분율, 직장실내 간접흡연 경험률은 현재 일을 하고 있는 비흡연자(과거 흡연자 포함)를 분모로 직장 실내에서 다른 사람이 피우는 담배 연기를 맡은 분율로 각각 산출하였다. 평균 음주빈도가 월1회 이상인 사람을 월간음주자, 폭음(한번의 술자리에서 남자 7잔(여자 5잔) 이상) 빈도가 월1회 이상인 사람을 월간폭음자로 정의하였다. 유산소신체활동실천율은 ‘한국인을 위한 신체활동 지침’에 따라 평소 일주일동안 중강도와 고강도 신체활동을 총 150분 이상 실천한 사람의 분율로 산출하였다. 일, 이동, 여가 시 활동 시간을 모두 합산하여 산출하였고, 고강도 신체활동 시간의 경우 응답 시간의 2배를 적용하여 산출하였다. 근력운동실천율은 종류나 시간에 관계없이 주 2회 이상 실천한 사람의 분율로 정의하였다.

건강생활실천율은 흡연, 음주, 신체활동 영역의 권고를 모두 실천(비흡연, 금주, 신체활동 실천)한 경우로 정의하였다. 비흡연은 평생 비흡연자이거나 과거 흡연하였더라도 현재 흡연하지 않는 경우, 금주는 비음주자이거나 최근 1년 동안 월 1회 미만 음주, 신체활동 실천은 유산소신체활동을 주 150분 이상 실천한 경우로 정의하여 산출하였다. 건강생활실천율 또한 조사에 적용한 신체활동 도구가 차이가 있었다(IPAQ: 2007년∼2013년, GPAQ: 2014년 이후).

**통계분석**

자료의 분석은 SAS 9.4 version 9.4 (SAS Institute Inc., Cary, N.C.)와 Joinpoint Regression Program version 4.1.1.1 (Suveillance Research Program, Statistical Methodology and Applications Branch, National Cancer Institute)를 이용하였다. 모든 결과는 대한민국에 거주하는 국민의 특성을 잘 대표할 수 있도록 가중치를 적용하여 복합표본설계분석방법으로 산출하였다. 흡연, 음주, 신체활동 관련 유병률의 성별, 소득수준별 추이는 연령 구조 차이에 따른 영향을 보정하기 위해 2005년 추계인구로 표준화하였고, 연령표준화율 및 표준오차는 SAS(PROC SURVEYREG)를 이용하여 계산하였다. 연령별 유병률은 조율값이며, SAS(PROC SURVEYMEANS)를 이용하여 계산하였다. SAS를 이용하여 산출된 유병률 및 표준오차를 미국 Joinpoint software에 적용하여 변곡점(joinpoint)을 0, 1개로 설정하여 모형을 추정하여 연간변화율(APCs and 95% confidence intervals)을 산출하였다. APCs는 유의수준 0.05하에서 연간변화율이 “0”인지 검증하였고, 최적 모형에 대한 통계적 유의성 검정에는 Monte Carlo method를 사용하였다.

**윤리 성명**

본 연구는 질병관리본부 연구윤리심의위원회를 통해서 연도별 계획에 따라 승인을 받았으며 (2018년 2018-01-03 등, 2007-2014년, 2018년) 일부 연도에는 생명윤리법 제2조제1호 및 동 시행규칙 제2조제2항제1호에 따라 심의면제를 받았다 (2015-2017년).

**RESULTS**

국민건강영양조사(1998-2018) 대상자의 일반적 특성은 Table 1과 같다. 조사대상은 만19세이상 96,408명이었으며, 남자 42,071명, 여자 54,337명으로 여자의 비율이 높았다. 연령별 참여분포를 살펴보면 20∼40대는 감소하였으며 50세 이상에서는 증가하였다.

남자의 현재흡연율은 1998년 66.3%에서 2018년 36.7%로 크게 감소한 반면(APC=-2.8, p<0.001), 여자의 현재흡연율은 6.5%에서 7.5%로 소폭 증가하였으나 통계적으로 유의하지는 않았다(APC=0.6) (Table 2). 남자의 경우 전 연령에서, 모든 소득수준 그룹에서 유의하게 감소하였으나(p<0.001), 소득수준 상-하 그룹의 흡연율 격차는 2010년 이후 줄어들지 않은 채 매년 약 10%p(2018년 기준, 31.0%, 40.1%)의 차이를 보였다. 반면, 여자는 60세이상에서는 감소, 20∼40대에서는 증가하였다(p<0.001). 현재 비흡연자의 가정, 직장, 공공장소에서의 간접흡연노출은 큰 폭으로 감소하여, 감소 폭은 각각 가정 실내 14.5%p(2005년 18.5%→2018년 4.0%), 직장 실내 25.4%p(2008년 36.9%→2018년 11.5%), 공공장소 실내 41.1%p(2013년 58.0%→2018년 16.9%)이었다(p<0.001) (Table 3).

월간음주율은 2005년에서 2018년까지 남자에서는 변화가 없었던 반면, 여자는 2005년 37.0%에서 2018년 51.2%로 증가하였다(APC=2.0, p<0.001) (Table 4). 여자의 월간음주율은 70세 이상을 제외한 전체 연령에서 유의하게 증가하였으며(p<0.001), 소득수준별로도 모든 군에서 증가하였다(p<0.001). 남자의 월간폭음률은 2005년 55.3%에서 2018년 50.8%로 감소하였으며 특히 20대의 감소 폭이 컸다(APC=-1.4, p<0.001) (Table 5). 반면 여자의 경우 월간폭음률이 2005년 17.2%에서 2018년 26.9%로 증가하였으며(APC=2.4, p<0.001), 연령별로 70세 이상을 제외한 모든 연령에서 증가하였다. 특히 20대의 경우 2005년 30.7%에서 2018년 49.8%로 증가하여 증가 폭(19.1%p)이 컸으며, 소득수준별로는 모든 그룹에서 유의하게 증가하였다(p<0.001).

유산소신체활동실천율은 남자는 2007년 76.3%에서 2018년 51.0%(APC=-4.2, p<0.001), 여자는 2007년 68.9%에서 2018년 44.0%(APC=-4.7, p<0.001)로, 남녀 모두 약 25%p 감소하였다 (Table 6). 특히 2014년 이후에는 남녀 모두 전 연령대에서 유의하게 감소하였으며, 소득수준별로도 모든 군에서 유의하게 감소하였다(p<0.001). 근력운동실천율은 남자는 2005년부터 2018년까지 변화가 없었던 반면, 여자는 2005년 11.2%에서 2018년 14.9%로 증가하였으며(APC=2.3, p<0.001), 60-70대의 증가 경향이 뚜렷하였다(2005년 60대 2.7%, 70대 1.4%→2018년 60대 14.3%, 70대 7.3%). 또한, 소득수준 상-하 그룹간 차이가 있어 상 그룹의 실천율이 하 그룹에 비해 2018년 기준으로 남자는 17.3%p, 여자는 6.5%p 높았다 (Data not shown).

건강생활실천율은 2007년 25.1%에서 2018년 14.5%로 약 10%p 감소하였다(APC=-5.3, p<0.001) (Table 7). 남녀 모두 감소하였으나 남자는 50세 이상에서, 여자는 전 연령에서 유의한 감소를 보였다. 건강생활실천율의 변화는 남자는 20∼30대의 흡연 및 음주 행태의 개선으로 감소 폭이 낮은 반면, 여자는 대부분 연령에서의 음주 및 신체활동 행태의 악화에 의해 2007년 대비 19.0%p 감소하였으며(APC=-6.4, p<0.001), 2014년을 기준으로 비교 시 8.8%p 감소하였다.

**DISCUSSION**

국민건강영양조사 자료를 이용하여 우리나라 성인의 주요 건강행태 변화를 살펴본 결과, 흡연율과 간접흡연 노출률은 지속적으로 감소하였고, 신체활동 실천율 또한 감소하였다. 음주의 경우 남자는 큰 변화가 없었던 반면, 여자에서 음주율과 폭음률 모두 증가하였다(Supplementary material).

1980년대 후반부터 민간에서 추진한 금연운동을 통해 흡연의 위해 및 금연의 필요성에 대한 인식 개선이 이루어져왔고 [11], 정부도 1995년 국민건강증진법을 통한 담배규제의 법적 근거 마련, 2005년 세계보건기구 담배규제기본협약(WHO Framework Convention on Tobacco Control) 비준 및 이행을 위한 정책(법적, 제도적 기반 강화, 금연캠페인 및 금연지원서비스 제공, 금연구역 확대 등)을 추진하여 왔다 [12-15]. 이러한 노력에 의해 2013년∼14년 남자의 현재흡연율은 약 43% 정도로 1998년에 비해 20%p 이상 감소하였다. 이후 2015년 담뱃값 인상, 대중매체 금연 캠페인 및 금연지원서비스 확대, 2016년 담뱃갑 경고그림 도입 등을 지속적으로 추진하여 [14-16], 2018년 남자의 현재흡연율은 36.7%까지 감소하였다. 또한 2012년 이후 간접흡연의 폐해 예방을 목적으로 시행된 공중 이용시설 전면 금연 시행, 일반 및 휴게음식점 등 실내 금연구역의 지속적 확대 등의 성과로 간접흡연 노출률(가정, 직장, 공공장소)은 1998년과 비교 시 약 1/3 이하로 감소하였다. 그러나 이러한 감소 경향에도 불구하고 남자의 현재흡연율은 미국(16.9%, 18세이상, 2015∼7년), 영국(16.5%, 18세이상, 2018년), 일본(17.8%, 20세이상, 2018년) 등 다른 나라와 비교 시 여전히 2배 이상 높은 수준이고 [17-19], 최근의 감소세 둔화 경향, 궐련 이외의 담배제품 사용 증가, 국민건강증진종합계획 2020의 목표치(29%)와 큰 차이가 있는 상황 등을 고려 시 보다 강력한 정책의 지속적인 추진이 필요하다. 특히, 2018년 궐련형, 액상형 전자담배의 사용은 조사항목으로 도입한 2013년에 비해 5배 정도 증가하였고, 궐련과 전자담배 중복 사용률 또한 22.5%까지 증가하였다. 이는 신종담배 유입으로 인한 담배제품 사용 증가에 대비한 선제적 정책 지원을 위해 신종담배 유해성 연구 및 사용행태 모니터링을 보다 적극적으로 수행할 필요가 있음을 시사한다.

여자의 흡연율은 남자와 다르게 다른 나라에 비해서는 낮은 수준이나 [9, 10, 17], 20∼40대에서 증가하는 경향을 보였다. 여자의 사회진출 증가, 여자에게 보다 친화적인 가향담배나 저독성 담배 출시 [20], 여자 흡연에 대한 사회적 낙인 효과가 줄어듦에 따른 과소 보고 감소 등이 여자 흡연자 증가에 기여요인으로 제시되고 있다 [21]. 현재까지 흡연율이 높은 남자 위주의 금연지원서비스와 관련 정책을 수행해 온 것을 고려할 때, 여자 흡연자의 흡연 행태 및 관련요인에 관한 질적 연구 등 심층 연구를 수행하여 여자 흡연자에 효과적인 금연 프로그램 개발이 필요하다 [22].

남자의 월간음주율 및 폭음률은 큰 변화없이 각각 70%, 50%이었고, 지표 정의가 조금차이가 있어 정확한 비교는 어렵지만 다른 나라에 비해 높은 수준이었다 [23]. 흡연과 다르게 음주율 및 폭음율이 높은 이유는 현재까지 주로 교육과 홍보, 고위험음주 및 알코올중독자 선별 및 치료 위주로 진행되어 주류 접근성을 크게 낮추지 못한 점이 관련이 있는 것으로 보고되고 있다 [24, 25]. 현재 공공장소에서의 음주 제한, 미디어 음주장면 가이드라인 제정, 주류광고 관련 규제 추진 등 음주 폐해 예방을 위한 정책들이 강화되고 있어 음주 추이 변화를 지속적으로 관찰할 필요가 있다 [26-28]. 여자의 경우 월간음주율 및 폭음률 모두 유의하게 증가하였고, 특히 20∼40대에서 크게 증가하였다. 여자 폭음률의 증가는 흡연과 마찬가지로 사회진출 증가, 저도주, 과일맛 소주 등 여자에게 친화적인 주종 개발 및 보급 확대에 의한 접근성 향상, 여자 음주자에 대한 부정적 인식의 완화 등의 영향으로 설명할 수 있다 [20]. 미국의 경우에도 고위험음주율, 알코올 사용장애자 비율이 남자에 비해 증가하였으며, 우리나라와 유사하게 여자의 교육 및 사회진출 기회 증가, 음주에 관해 수용적인 사회 분위기로의 변화 등과 관련이 있다고 제언하였다 [23, 29, 30].

유산소신체활동 실천율은 지속적으로 감소하는 경향으로 2018년 남자 51%, 여자 44%였다. 영역별(일, 이동, 여가활동)로 구분하여 감소 경향을 분석한 결과, 일과 관련된 신체활동 비실천(2014년 97.4%, 2018년 98.6%), 여가와 관련된 신체활동 비실천(2014년 90.2%, 2018년 91.7%)은 큰 변화가 없는 반면 이동 시 신체활동 비실천(2014년 38.5%, 2018년 45.1%)은 6.6%p 증가하여, 유산소신체활동 실천율 감소는 이동 시 신체활동 감소에 의한 것으로 보인다. 신체활동 실천은 개인적, 사회경제적, 환경적 요인과 밀접하게 관련이 있어 신체활동 증가를 위해서 지식 제공 및 동기 부여, 환경 조성에 의한 접근성 향상이 필요하다 [31-34]. 2013년부터‘한국인을 위한 신체활동 지침’을 발표하여 신체활동의 실천을 강조하고, 지역사회통합건강증진사업 중 신체활동 개별사업을 추진 중임에도 신체활동 지표의 개선이 없어 심층분석에 의한 원인 파악 후 실천율 향상을 위한 방안 마련이 필요할 것으로 보인다.

건강생활(비흡연, 금주, 유산소신체활동) 실천율은 2007년 25.1%에서 2018년 14.5%로 10.6%p 감소하였고, 특히 여자의 실천율이 크게 감소하였다. 남자의 경우 흡연, 음주 행태 개선으로 특히, 남자 20대의 경우 건강생활 실천율이 5.1%p 증가한 반면(2014년 12.3%, 2018년 17.4%), 여자의 건강행태는 전반적으로 악화되었으며 이는 모든 연령, 소득수준에서 건강생활 실천율의 감소로 나타났다. 금연, 절주, 신체활동실천 등 건강행태는 전체 사망, 암 및 심뇌혈관질환으로 인한 사망 위험의 감소와 건강수명 연장과 관련이 있었다 [8-10, 35-37]. 본 연구에서 바람직한 식생활, 건강체중을 제외한 건강생활(비흡연, 금주, 유산소신체활동실천)을 실천하는 정도가 남자는 10명 중 1명, 여자는 10명중 2명으로 매우 낮아 개별 건강행태 개선을 위한 정책이나 사업을 시급하게 추진할 필요성을 뒷받침해 주고 있다. 본 연구는 만성질환의 주요 위험요인인 건강행태의 20년간의 변화를 파악하고자 하였으나 음주와 신체활동의 경우 측정도구 도입 및 변경으로 인해 흡연과 달리 20년간의 추이를 분석하지 못한 제한점이 있다.

지난 20년간 국민건강영양조사 결과, 흡연행태(남자)를 제외하고서는 신체활동 감소, 음주 및 폭음 증가(여자) 등 건강행태는 개선되고 있지 않았다. 이는 같은 기간 동안의 비만, 이상지질혈증, 당뇨병 등 만성질환 유병의 증가와 관련이 있을 것으로 사료된다. 향후 만성질환 증가, 이로 인한 질병부담 감소를 위해 건강행태 개선을 위한 효과적인 프로그램 수행이 필요하다.

**CONFLICT OF INTEREST**

모든 저자는 본 연구에 대해 표명할 이해상충이 없음

**FUNDING**

없음

**ACKNOWLEDGEMENTS**

지난 20년동안 국민건강영양조사에 참여해주신 대상자께 감사합니다. 또한, 조사를 전담하여 수행한 조사수행팀, 조사 지원과 자문을 주신 관련학회 및 전문가 자문단에게도 감사합니다.

**AUTHOR CONTRIBUTIONS**

Conceptualization: SK, KO. Data curation: SK, JK, SC. Formal analysis: SP. Funding acquisition: None. Project administration: YK, OP, KO. Visualization: SK, SP. Writing – original draft: SK, SP, KO. Writing – review & editing: SC, JK, YK, OP, KO.

**ORCID**

Soyeon Kim: *https://orcid.org/0000-0002-5027-1808*; Sunhye Choi: *https://orcid.org/0000-0002-2942-0290*; Jihee Kim: *https:// orcid.org/0000-0002-0212-8177*; Suyeon Park: *https://orcid.org/ 0000-0001-8134-8436*; Youngtaek Kim: *https://orcid.org/0000-0003-0139-7620*; Ok Park: *https://orcid.org/0000-0002-9477-9523*; Kyungwon Oh: *https://orcid.org0000-0001-8097-6078*

**REFERENCES**

1. GBD 2017 Risk Factor Collaborators. Global, regional, and national comparative risk assessment of 84 behavioural, environmental and occupational, and metabolic risks or clusters of risks for 195 countries and territories, 1990-2017: a systematic analysis for the Global Burden of Disease Study 2017. Lancet 2018;392:1923-1994.

2. Korea Centers for Disease Control and Prevention. Factbook: non-communicable diesease 2019. Cheongju: Korea Centers for Disease Control and Prevention; 2020, p. 6 (Korean).

3. Virani SS, Alonso A, Benjamin EJ, Bittencourt MS, Callaway CW, Carson AP, et al. Heart disease and stroke statistics-2020 update: a report from the American Heart Association. Circulation 2020;141:e139-e596..

4. GBD 2015 Tobacco Collaborators. Smoking prevalence and attributable disease burden in 195 countries and territories, 1990-2015: a systematic analysis from the Global Burden of Disease Study 2015. Lancet 2017;389:1885-1906.

5. Room R, Babor T, Rehm J. Alcohol and public health. Lancet 2005;365:519-530.

6. Coste J, Quinquis L, D’Almeida S, Audureau E. Smoking and health-related quality of life in the general population. Independent relationships and large differences according to patterns and quantity of smoking and to gender. PLoS One 2014;9:e91562.

7. Al-Rubaye AK, Johansson K, Alrubaiy L. The association of health behavioral risk factors with quality of life in northern Sweden-a cross-sectional survey. J Gen Fam Med 2020;21:167-177.

8. Li Y, Pan A, Wang DD, Liu X, Dhana K, Franco OH, et al. Impact of healthy lifestyle factors on life expectancies in the US population. Circulation 2018;138:345-355.

9. Yun JE, Won S, Kimm H, Jee SH. Effects of a combined lifestyle score on 10-year mortality in Korean men and women: a prospective cohort study. BMC Public Health 2012;12:673.

10. Fransen HP, May AM, Beulens JW, Struijk EA, de Wit GA, Boer JM, et al. Association between lifestyle factors and quality-adjusted life years in the EPIC-NL cohort. PLoS One 2014;9:e111480.

11. Korean Association of Smoking and Health. The war changes the world: understanding the smoking cessation movement and tobacco control policy, Seoul: Korean Association of Smoking and Health; 2018, p. 34-57 (Korean, author’s translation).

12. Levy DT, Cho SI, Kim YM, Park S, Suh MK, Kam S. SimSmoke model evaluation of the effect of tobacco control policies in Korea: the unknown success story. Am J Public Health 2010;100:1267-1273.

13. Park J, Minh LN, Shin SH, Oh JK, Yun EH, Lee D, et al. Influence of new tobacco control policies and campaigns on Quitline call volume in Korea. Tob Induc Dis 2019;17:21.

14. Korea Health Promotion Institute. Changes and development of the smoking prevention campaign in Korea; 2019 [cited 2020 Sep 20]. Available from https://nosmk.khealth.or.kr/nsk/user/extra/ntcc/nosmokeFile/fileView/jsp/Page.do?siteMenuIdx=94&fileNo=581&spage=1&sRow=12&dataNo=8&dataGr=4&datalv=0&searchFile=&listRange=makeYM&listType=album&level1Idx=8&level2Idx=31&postIdx=625 (Korean, author’s translation).

15. Korea Health Promotion Institute. Non-smoking zone policy and future tasks in Korea; 2019 [cited 2020 Sep 20]. Available from https://nosmk.khealth.or.kr/nsk/user/extra/ntcc/nosmokeFile/fileView/jsp/Page.do?siteMenuIdx=94&fileNo=560&spage=1&sRow=12&dataNo=8&dataGr=4&datalv=0&searchFile=&listRange=makeYM&listType=album&level1Idx=8&level2Idx=31&postIdx=601 (Korean, author’s translation).

16. Kwon DS, Kim TH, Byun MK, Kim HJ, Lee HS, Park HJ, et al. Positive effects of the national cigarette price increase policy on smoking cessation in South Korea. Tuberc Respir Dis (Seoul) 2020;83:71-80.

17. Centers for Disease Control and Prevention. Health United States, 2018-data finder; 2019 [cited 2020 Sep 21]. Available from: https://www.cdc.gov/nchs/hus/contents2018.htm#Table_019.

18. Office of National Statistics. Adult smoking habits in the UK: 2018; 2019 [cited 2020 Sep 21]. Available from: https://www.ons.gov.uk/peoplepopulationandcommunity/healthandsocialcare/healthanEpidemiolHealth 2021;43:e202102612 | www.e-epih.orgdlifeexpectancies/bulletins/adultsmokinghabitsingreatbritain/2018.

19. National Institute of Health and Nutrition of Japan. National Health and Nutrition Survey [cited 2020 Sep 19]. Available from https://www.nibiohn.go.jp/eiken/kenkounippon21/en/eiyouchousa/kekka_eiyou_chousa.html (Japanese).

20. Korea Centers for Disease Control and Prevention. Trend analysis and association study of chronic diseases and risk factors: findings from the KNHANES 1998-2018. Cheongju; Korea Centers for Disease Control and Prevention; 2020, p. 34-35 (Korean).

21. Jung-Choi KH, Khang YH, Cho HJ. Hidden female smokers in Asia: a comparison of self-reported with cotinine-verified smoking prevalence rates in representative national data from an Asian population. Tob Control 2012;21:536-542.

22. Lee Y, Kim KH, Kang H, Cho S, Kim SJ, Kim M, et al. An evidence-based literature review and evaluation of tobacco control policy in Korea: policy implications for young adults. Health Soc Welf Rev 2020;40:616-650 (Korean).

23. World Health Organization. Global status report on alcohol and health 2018 [cited 2020 Sep 20]. Available from: https://www.who.int/publications/i/item/9789241565639.

24. Korea Centers for Disease Control and Prevention. Development of comprehensive assessment questionnaire for alcohol problem, and a pilot survey. Cheongju: Korea Centers for Disease Control and Prevention; 2015, p. 36-38, 147-148 (Korean).

25. Jekarl J, Kim KK, Yoo S, Choi SA, Kim T, Ju M. Status and challenges of policies to reduce heavy drinking among women based on monitoring of Health Plan 2020. Korean J Health Educ Promot 2017;34:27-39 (Korean).

26. Korea Health Promotion Institute. Media guidelines for drinking scene; 2017 [cited 2020 Sep 20]. Available from https://www.khealth.or.kr/kps/publish/view?menuId=MENU00890&page_no=B2017003&board_idx=9965 (Korean, author’s translation).

27. Korea Centers for Disease Control and Prevention. Factor analysis of individual drinking behavior and development of guidelines for improving drinking behavior. Cheongju: Korea Centers for Disease Control and Prevention; 2018, p. 162-168 (Korean).

28. Ministry of Health and Welfare of Korea. Action plan for preventing alcohol harms; 2018 [cited 2020 Sep 20]. Available from: https://www.mohw.go.kr/react/al/sal0301vw.jsp?PAR_MENU_ID=04&MENU_ID=0403&page=1&CONT_SEQ=346622 (Korean).

29. Grant BF, Chou SP, Saha TD, Pickering RP, Kerridge BT, Ruan WJ, et al. Prevalence of 12-month alcohol use, high-risk drinking, and DSM-IV alcohol use disorder in the United States, 2001-2002 to 2012-2013: results from the National Epidemiologic Survey on Alcohol and Related Conditions. JAMA Psychiatry 2017;74:911-923.

30. Hasin DS, Shmulewitz D, Keyes K. Alcohol use and binge drinking among U.S. men, pregnant and non-pregnant women ages 18-44: 2002-2017. Drug Alcohol Depend 2019;205:107590.

31. Lee D. What determines physical activity of Korean adults? Nationwide cross-sectional survey study. Korea Health Promot Forum 2018;7:44-55 (Korean).

32. An KY. Physical activity level in Korean adults: the Korea National Health and Nutrition Examination Survey 2017. Epidemiol Health 2019;41:e2019047.

33. Moreno-Llamas A, García-Mayor J, De la Cruz-Sánchez E. Physical activity barriers according to social stratification in Europe. Int J Public Health 2020;65:1477-1484.

34. Pan SY, Cameron C, Desmeules M, Morrison H, Craig CL, Jiang X. Individual, social, environmental, and physical environmental correlates with physical activity among Canadians: a cross-sectional study. BMC Public Health 2009;9:21.

35. Lee I, Kim S, Kang H. Lifestyle risk factors and all-cause and cardiovascular disease mortality: data from the Korean longitudinal study of aging. Int J Environ Res Public Health 2019;16:3040.

36. Zhu N, Yu C, Guo Y, Bian Z, Han Y, Yang L, et al. Adherence to a healthy lifestyle and all-cause and cause-specific mortality in Chinese adults: a 10-year prospective study of 0.5 million people. Int J Behav Nutr Phys Act 2019;16:98.

37. Ford ES, Zhao G, Tsai J, Li C. Low-risk lifestyle behaviors and all-cause mortality: findings from the National Health and Nutrition Examination Survey III Mortality Study. Am J Public Health 2011;101:1922-1929.

**Table1. Characteristics of health interview subjects of the Korea National Health and Nutrition Examination Survey**

|  | | 1998 | | 2001 | | 2005 | | 2007 | | 2008 | | 2009 | | 2010 | | 2011 | | 2012 | | 2013 | | 2014 | | 2015 | | 2016 | | 2017 | | 2018 | |
| --- | --- | --- | --- | --- | --- | --- | --- | --- | --- | --- | --- | --- | --- | --- | --- | --- | --- | --- | --- | --- | --- | --- | --- | --- | --- | --- | --- | --- | --- | --- | --- |
|  | | n | % | n | % | n | % | n | % | n | % | n | % | n | % | n | % | n | % | n | % | n | % | n | % | n | % | n | % | n | % |
| Total (≥19 yr) | | 8,991 |  | 8,072 |  | 7,802 |  | 2,983 |  | 6,817 |  | 7,511 |  | 6,352 |  | 6,224 |  | 5,995 |  | 5,792 |  | 5,677 |  | 5,632 |  | 6,129 |  | 6,193 |  | 6,238 |  |
| Men | | 4,180 |  | 3,679 |  | 3,510 |  | 1,242 |  | 2,847 |  | 3,252 |  | 2,745 |  | 2,651 |  | 2,506 |  | 2,485 |  | 2,385 |  | 2,457 |  | 2,647 |  | 2,749 |  | 2,736 |  |
| Age (yr) | 19-29 | 886 | 21.2 | 715 | 19.4 | 583 | 16.6 | 141 | 11.4 | 367 | 12.9 | 485 | 14.9 | 308 | 11.2 | 296 | 11.2 | 265 | 10.6 | 320 | 12.9 | 259 | 10.9 | 338 | 13.8 | 308 | 11.6 | 344 | 12.5 | 359 | 13.1 |
|  | 30-39 | 1,091 | 26.1 | 944 | 25.7 | 812 | 23.1 | 247 | 19.9 | 600 | 21.1 | 597 | 18.4 | 531 | 19.3 | 459 | 17.3 | 416 | 16.6 | 435 | 17.5 | 412 | 17.3 | 315 | 12.8 | 462 | 17.5 | 409 | 14.9 | 420 | 15.4 |
|  | 40-49 | 888 | 21.2 | 911 | 24.8 | 872 | 24.8 | 248 | 20.0 | 581 | 20.4 | 646 | 19.9 | 542 | 19.7 | 471 | 17.8 | 435 | 17.4 | 506 | 20.4 | 413 | 17.3 | 417 | 17.0 | 510 | 19.3 | 504 | 18.3 | 479 | 17.5 |
|  | 50-59 | 616 | 14.7 | 522 | 14.2 | 579 | 16.5 | 205 | 16.5 | 491 | 17.2 | 545 | 16.8 | 488 | 17.8 | 522 | 19.7 | 474 | 18.9 | 456 | 18.4 | 448 | 18.8 | 506 | 20.6 | 461 | 17.4 | 548 | 19.9 | 506 | 18.5 |
|  | 60-69 | 476 | 11.4 | 381 | 10.4 | 445 | 12.7 | 217 | 17.5 | 441 | 15.5 | 542 | 16.7 | 481 | 17.5 | 471 | 17.8 | 461 | 18.4 | 412 | 16.6 | 434 | 18.2 | 475 | 19.3 | 451 | 17.0 | 475 | 17.3 | 503 | 18.4 |
|  | 70+ | 223 | 5.3 | 206 | 5.6 | 219 | 6.2 | 184 | 14.8 | 367 | 12.9 | 437 | 13.4 | 395 | 14.4 | 432 | 16.3 | 455 | 18.2 | 356 | 14.3 | 419 | 17.6 | 406 | 16.5 | 455 | 17.2 | 469 | 17.1 | 469 | 17.1 |
| Household income^1^ | Low | 826 | 19.8 | 582 | 16.7 | 712 | 20.5 | 237 | 19.9 | 554 | 20.1 | 639 | 19.9 | 533 | 19.6 | 531 | 20.3 | 488 | 19.8 | 500 | 20.3 | 477 | 20.1 | 483 | 19.8 | 534 | 20.3 | 545 | 19.9 | 543 | 19.9 |
|  | Low-Middle | 813 | 19.4 | 718 | 20.6 | 726 | 21.0 | 237 | 19.9 | 552 | 20.0 | 658 | 20.5 | 538 | 19.8 | 514 | 19.6 | 494 | 20.0 | 492 | 20.0 | 476 | 20.0 | 488 | 20.0 | 529 | 20.1 | 543 | 19.8 | 543 | 19.9 |
|  | Middle | 864 | 20.7 | 680 | 19.5 | 682 | 19.7 | 238 | 19.9 | 539 | 19.5 | 636 | 19.8 | 580 | 21.4 | 519 | 19.8 | 480 | 19.5 | 492 | 20.0 | 482 | 20.3 | 492 | 20.2 | 519 | 19.7 | 548 | 20.0 | 555 | 20.4 |
|  | Middle-High | 842 | 20.1 | 695 | 20.0 | 669 | 19.3 | 242 | 20.3 | 572 | 20.7 | 634 | 19.7 | 517 | 19.1 | 521 | 19.9 | 496 | 20.1 | 490 | 19.9 | 475 | 20.0 | 486 | 19.9 | 523 | 19.8 | 550 | 20.1 | 541 | 19.9 |
|  | High | 835 | 20.0 | 804 | 23.1 | 676 | 19.5 | 239 | 20.0 | 543 | 19.7 | 644 | 20.1 | 545 | 20.1 | 534 | 20.4 | 507 | 20.6 | 491 | 19.9 | 467 | 19.6 | 488 | 20.0 | 530 | 20.1 | 554 | 20.2 | 543 | 19.9 |
| Women | | 4,811 |  | 4,393 |  | 4,292 |  | 1,741 |  | 3,970 |  | 4,259 |  | 3,607 |  | 3,573 |  | 3,489 |  | 3,307 |  | 3,292 |  | 3,175 |  | 3,482 |  | 3,444 |  | 3,502 |  |
| Age (yr) | 19-29 | 1,073 | 22.3 | 911 | 20.7 | 753 | 17.5 | 198 | 11.4 | 527 | 13.3 | 565 | 13.3 | 469 | 13.0 | 403 | 11.3 | 399 | 11.4 | 408 | 12.3 | 379 | 11.5 | 355 | 11.2 | 393 | 11.3 | 384 | 11.1 | 405 | 11.6 |
|  | 30-39 | 1,122 | 23.3 | 1,054 | 24.0 | 930 | 21.7 | 388 | 22.3 | 816 | 20.6 | 833 | 19.6 | 730 | 20.2 | 666 | 18.6 | 601 | 17.2 | 589 | 17.8 | 553 | 16.8 | 454 | 14.3 | 627 | 18.0 | 506 | 14.7 | 496 | 14.2 |
|  | 40-49 | 916 | 19.0 | 961 | 21.9 | 1,006 | 23.4 | 317 | 18.2 | 749 | 18.9 | 853 | 20.0 | 641 | 17.8 | 618 | 17.3 | 587 | 16.8 | 631 | 19.1 | 568 | 17.3 | 570 | 18.0 | 633 | 18.2 | 634 | 18.4 | 655 | 18.7 |
|  | 50-59 | 722 | 15.0 | 595 | 13.5 | 650 | 15.1 | 300 | 17.2 | 644 | 16.2 | 707 | 16.6 | 697 | 19.3 | 690 | 19.3 | 663 | 19.0 | 640 | 19.4 | 634 | 19.3 | 658 | 20.7 | 649 | 18.6 | 667 | 19.4 | 692 | 19.8 |
|  | 60-69 | 590 | 12.3 | 497 | 11.3 | 525 | 12.2 | 273 | 15.7 | 640 | 16.1 | 668 | 15.7 | 563 | 15.6 | 577 | 16.1 | 593 | 17.0 | 495 | 15.0 | 540 | 16.4 | 551 | 17.4 | 568 | 16.3 | 626 | 18.2 | 606 | 17.3 |
|  | 70+ | 388 | 8.1 | 375 | 8.5 | 428 | 10.0 | 265 | 15.2 | 594 | 15.0 | 633 | 14.9 | 507 | 14.1 | 619 | 17.3 | 646 | 18.5 | 544 | 16.5 | 618 | 18.8 | 587 | 18.5 | 612 | 17.6 | 627 | 18.2 | 648 | 18.5 |
| Household income | Low | 935 | 19.4 | 753 | 18.3 | 887 | 20.9 | 324 | 19.6 | 776 | 20.2 | 839 | 19.9 | 709 | 20.0 | 715 | 20.2 | 687 | 20.0 | 647 | 19.7 | 665 | 20.3 | 622 | 19.7 | 688 | 19.8 | 685 | 20.0 | 702 | 20.1 |
|  | Low-Middle | 946 | 19.7 | 826 | 20.0 | 823 | 19.4 | 333 | 20.2 | 778 | 20.2 | 852 | 20.2 | 726 | 20.5 | 701 | 19.8 | 699 | 20.4 | 648 | 19.8 | 646 | 19.8 | 634 | 20.1 | 699 | 20.1 | 691 | 20.2 | 702 | 20.1 |
|  | Middle | 952 | 19.8 | 784 | 19.0 | 838 | 19.7 | 330 | 20.0 | 758 | 19.7 | 844 | 20.0 | 706 | 19.9 | 701 | 19.8 | 671 | 19.6 | 672 | 20.5 | 646 | 19.8 | 636 | 20.2 | 703 | 20.3 | 683 | 19.9 | 700 | 20.0 |
|  | Middle-High | 1,010 | 21.0 | 836 | 20.3 | 854 | 20.1 | 330 | 20.0 | 771 | 20.1 | 867 | 20.6 | 706 | 19.9 | 705 | 19.9 | 685 | 20.0 | 663 | 20.2 | 663 | 20.3 | 632 | 20.0 | 697 | 20.1 | 686 | 20.0 | 691 | 19.8 |
|  | High | 968 | 20.1 | 922 | 22.4 | 848 | 20.0 | 334 | 20.2 | 759 | 19.8 | 814 | 19.3 | 702 | 19.8 | 712 | 20.1 | 689 | 20.1 | 651 | 19.8 | 649 | 19.9 | 630 | 20.0 | 683 | 19.7 | 683 | 19.9 | 697 | 20.0 |

Values are presented as the number

^1^Calculated as monthly household income divided by the square root of the number of persons in the household, categorized into quantile by age and gender

**Table 2. Trends in the prevalence of current cigarette smoking**^1^

|  | | 1998 | 2001 | 2005 | 2007 | 2008 | 2009 | 2010 | 2011 | 2012 | 2013 | 2014 | 2015 | 2016 | 2017 | 2018 | Difference (1998 to 2018) | APC^3^ | APC and year of any significant change in trend slope | | |
| --- | --- | --- | --- | --- | --- | --- | --- | --- | --- | --- | --- | --- | --- | --- | --- | --- | --- | --- | --- | --- | --- |
|  |  |  |  |  |  |  |  |  |  |  |  |  |  |  |  |  |  |  | before | change year | after |
| Total (≥19 yr) | | 35.1 | 30.2 | 28.8 | 25.3 | 27.8 | 27.3 | 27.5 | 27.1 | 25.8 | 24.1 | 24.2 | 22.6 | 23.9 | 22.3 | 22.4 | -12.7 | **-2.1*** | **-2.4*** | 2005 | **-1.9*** |
| Men | | 66.3 | 60.9 | 51.7 | 45.1 | 47.8 | 47.0 | 48.3 | 47.3 | 43.7 | 42.2 | 43.2 | 39.4 | 40.7 | 38.1 | 36.7 | -29.6 | **-2.8*** | **-3.4*** | 2005 | **-2.4*** |
| Age (yr) | 19-29 | 70.7 | 64.7 | 55.5 | 47.5 | 53.6 | 51.9 | 47.3 | 44.9 | 41.5 | 37.0 | 34.8 | 38.7 | 41.7 | 37.3 | 34.9 | -35.8 | **-3.4*** | **-3.1*** | 2008 | **-3.9*** |
|  | 30-39 | 71.6 | 67.9 | 60.2 | 58.3 | 56.4 | 56.2 | 60.9 | 63.7 | 54.8 | 54.5 | 53.2 | 48.0 | 51.5 | 42.7 | 39.9 | -31.7 | **-2.0*** | **-1.8*** | 2016 | -12.9 |
|  | 40-49 | 67.9 | 66.7 | 55.2 | 48.8 | 49.1 | 48.9 | 53.6 | 47.0 | 49.5 | 48.0 | 54.4 | 45.8 | 43.9 | 46.3 | 44.1 | -23.8 | **-2.3*** | **-3.1*** | 2008 | -1.1 |
|  | 50-59 | 62.2 | 55.5 | 47.7 | 34.0 | 41.5 | 41.6 | 45.0 | 44.4 | 41.8 | 40.8 | 39.4 | 36.5 | 38.2 | 37.0 | 40.6 | -21.6 | **-2.5*** | **-4.0*** | 2007 | **-1.1*** |
|  | 60-69 | 58.2 | 49.9 | 38.3 | 31.1 | 34.5 | 33.8 | 30.8 | 32.5 | 26.9 | 32.5 | 35.8 | 26.1 | 25.7 | 26.6 | 26.7 | -31.5 | **-4.0*** | **-5.6*** | 2007 | **-2.3*** |
|  | 70+ | 50.5 | 33.8 | 27.5 | 23.3 | 27.9 | 23.7 | 24.7 | 28.8 | 23.2 | 15.6 | 19.4 | 17.0 | 18.0 | 18.2 | 14.7 | -35.8 | **-5.3*** | -7.0 | 2005 | **-4.3*** |
| Household income^2^ | Low | 70.0 | 68.7 | 58.8 | 50.4 | 55.9 | 53.4 | 53.1 | 54.3 | 48.7 | 47.9 | 46.3 | 41.8 | 40.3 | 42.8 | 40.1 | -29.9 | **-2.7*** | **-2.4*** | 2011 | **-3.7*** |
|  | Low-Middle | 68.7 | 61.6 | 53.9 | 47.3 | 49.1 | 49.9 | 52.2 | 50.0 | 48.0 | 44.1 | 42.7 | 38.3 | 44.0 | 43.4 | 41.8 | -26.9 | **-2.6*** | **-3.4*** | 2005 | **-2.0*** |
|  | Middle | 65.1 | 62.4 | 52.4 | 46.9 | 47.6 | 47.8 | 45.8 | 47.0 | 39.7 | 43.8 | 46.4 | 42.2 | 39.8 | 37.3 | 35.4 | -29.7 | **-2.8*** | **-2.7*** | 2016 | -5.8 |
|  | Middle-High | 65.2 | 57.1 | 47.5 | 45.6 | 47.5 | 44.7 | 46.3 | 41.7 | 39.5 | 38.6 | 42.7 | 40.0 | 43.4 | 38.5 | 34.4 | -30.8 | **-2.8*** | **-4.2*** | 2005 | **-1.9*** |
|  | High | 63.7 | 54.5 | 45.5 | 34.5 | 38.8 | 38.4 | 42.6 | 42.7 | 41.2 | 37.2 | 37.6 | 33.9 | 36.5 | 28.0 | 31.0 | -32.7 | **-3.4*** | **-4.5*** | 2007 | **-2.1*** |
| Women | | 6.5 | 5.2 | 5.7 | 5.3 | 7.4 | 7.1 | 6.3 | 6.8 | 7.9 | 6.2 | 5.7 | 5.5 | 6.4 | 6.0 | 7.5 | 1.0 | 0.6 | 1.5 | 2009 | -0.5 |
| Age (yr) | 19-29 | 5.1 | 4.4 | 6.0 | 7.6 | 12.7 | 11.1 | 7.4 | 10.4 | 13.6 | 9.1 | 8.9 | 6.9 | 7.2 | 9.7 | 10.9 | 5.8 | **3.4*** | **8.4*** | 2009 | -1.9 |
|  | 30-39 | 4.5 | 3.6 | 4.4 | 4.4 | 7.1 | 7.9 | 7.6 | 8.9 | 9.0 | 6.9 | 7.0 | 6.7 | 7.6 | 6.8 | 8.3 | 3.8 | **3.6*** | **6.2*** | 2011 | -1.9 |
|  | 40-49 | 4.4 | 3.7 | 5.7 | 4.5 | 5.7 | 5.5 | 6.6 | 4.1 | 5.5 | 6.2 | 5.0 | 4.9 | 5.6 | 5.7 | 8.7 | 4.3 | **2.3*** | 1.2 | 2016 | 20.5 |
|  | 50-59 | 7.2 | 4.0 | 6.7 | 4.6 | 3.4 | 4.1 | 5.2 | 5.0 | 7.9 | 3.7 | 2.5 | 5.4 | 7.1 | 3.3 | 5.0 | -2.2 | -1.1 | -3.5 | 2008 | 1.0 |
|  | 60-69 | 12.0 | 6.2 | 3.5 | 4.6 | 4.7 | 4.5 | 2.9 | 3.8 | 1.6 | 4.0 | 2.5 | 2.8 | 4.0 | 2.8 | 3.6 | -8.4 | **-6.9*** | **-14.3*** | 2005 | -2.0 |
|  | 70+ | 14.5 | 18.0 | 9.3 | 6.6 | 8.7 | 6.8 | 5.0 | 5.1 | 3.2 | 3.1 | 3.8 | 3.7 | 3.4 | 1.9 | 1.1 | -13.4 | -10.7* | **-9.9*** | 2016 | -39.1 |
| Household income | Low | 10.2 | 7.9 | 8.6 | 11.3 | 9.9 | 11.3 | 10.6 | 11.9 | 11.4 | 9.3 | 11.2 | 9.4 | 8.9 | 9.3 | 10.7 | 0.5 | 0.6 | 1.5 | 2011 | -1.7 |
|  | Low-Middle | 6.7 | 5.1 | 6.0 | 7.3 | 8.4 | 7.3 | 5.7 | 8.6 | 10.6 | 7.8 | 5.9 | 5.9 | 8.2 | 5.3 | 10.6 | 3.9 | 1.7 | 2.4 | 2012 | -0.6 |
|  | Middle | 5.9 | 3.0 | 4.8 | 2.7 | 7.8 | 5.9 | 3.6 | 4.9 | 6.5 | 5.5 | 4.2 | 4.7 | 5.2 | 7.0 | 6.9 | 1.0 | 1.0 | -0.1 | 2015 | 11.3 |
|  | Middle-High | 5.7 | 3.9 | 4.3 | 2.1 | 4.6 | 6.6 | 6.4 | 3.9 | 4.2 | 4.4 | 4.1 | 4.2 | 4.5 | 4.6 | 5.6 | -0.1 | -0.4 | -2.9 | 2005 | 0.9 |
|  | High | 4.9 | 5.7 | 4.9 | 2.7 | 6.5 | 3.8 | 4.9 | 3.9 | 5.8 | 4.3 | 2.4 | 3.6 | 5.3 | 3.4 | 3.2 | -1.7 | -1.7 | 0.0 | 2008 | -3.6 |

Values are presented as weighted %; Age-standardized prevalence was calculated using the 2005 population projections for Korea

^1^Current cigarette smoking: percentage of adults who have smoked at least 100 cigarettes during their lifetime and who are currently smokers

^2^Calculated as monthly household income divided by the square root of the number of persons in the household, categorized into quantile by age and gender

^3^The annual percent change (APC) is significantly different from 0

* p < 0.001

**Table 3. Trends in the prevalence of exposure to secondhand smoking**

|  | 2005 | 2007 | 2008 | 2009 | 2010 | 2011 | 2012 | 2013 | 2014 | 2015 | 2016 | 2017 | 2018 | Difference (2005 to 2018) | APC^4^ | APC and year of any significant change in trend slope | | |
| --- | --- | --- | --- | --- | --- | --- | --- | --- | --- | --- | --- | --- | --- | --- | --- | --- | --- | --- |
|  |  |  |  |  |  |  |  |  |  |  |  |  |  |  |  | before | change year | after |
| At home (≥19 yr) ^1^ | | | | | | | | | | | | | | | | | | |
| Total | 18.5 | 14.7 | 15.5 | 14.9 | 14.9 | 12.5 | 11.9 | 10.9 | 10.7 | 8.2 | 6.4 | 4.7 | 4.0 | -14.5 | **-8.8*** | **-5.9*** | 2014 | **-22.7*** |
| Men | 7.1 | 4.4 | 6.5 | 6.9 | 5.8 | 4.9 | 4.8 | 5.5 | 4.8 | 4.2 | 4.0 | 2.3 | 1.0 | -6.1 | **-7.3*** | **-4.6*** | 2016 | **-50.1*** |
| Women | 24.1 | 20.5 | 20.5 | 19.4 | 19.8 | 16.7 | 16.0 | 14.1 | 13.9 | 10.7 | 7.9 | 6.3 | 6.1 | -18.0 | **-8.7*** | **-6.1*** | 2014 | **-21.0*** |
| Workplace (≥19 yr) ^2^ | | | | | | | | | | | | | | | | | | |
| Total | 36.9 | 46.0 | 45.4 | 45.8 | 49.2 | 45.2 | 46.1 | 47.4 | 40.1 | 26.9 | 17.4 | 12.7 | 11.5 | -25.4 | **-5.3*** | **2.7*** | 2013 | **-26.9*** |
| Men | 44.6 | 55.2 | 53.4 | 53.3 | 58.7 | 55.3 | 54.5 | 57.3 | 49.1 | 36.2 | 23.5 | 17.3 | 14.4 | -30.2 | -3.9 | **2.9*** | 2013 | **-25.1*** |
| Women | 31.8 | 36.4 | 38.2 | 39.5 | 41.8 | 37.2 | 38.9 | 38.7 | 32.8 | 18.5 | 12.1 | 8.5 | 8.7 | -23.1 | **-5.4*** | 2.3 | 2013 | **-29.9*** |
| Public places (≥19 yr) ^3^ | | | | | | | | | | | | | | | | | | |
| Total | - | - | - | - | - | - | - | 58.0 | 52.2 | 35.4 | 22.3 | 21.1 | 16.9 | -41.1 | **-23.2*** | **-24.7*** | 2016 | -19.4 |
| Men | - | - | - | - | - | - | - | 62.6 | 56.3 | 41.0 | 26.9 | 26.0 | 19.1 | -43.5 | **-21.3*** | **-19.3*** | 2015 | **-23.2*** |
| Women | - | - | - | - | - | - | - | 55.4 | 49.8 | 31.8 | 19.4 | 17.9 | 15.4 | -40 | **-24.6*** | **-26.0*** | 2016 | -20.8 |

Values are presented as weighted %; Age-standardized prevalence was calculated using the 2005 population projections for Korea

^1^Secondhand smoking at home: percentage of current non-smokers who have been exposed to secondhand smoke at home during the past 7 days

^2^Secondhand smoking in the workplace: percentage of current non-smokers who have been exposed to secondhand smoke in the workplace during the past 7 days

^3^Secondhand smoking in public places: percentage of current non-smokers who have been exposed to secondhand smoke in public places during the past 7 days

^4^The annual percent change (APC) is significantly different from 0

* p < 0.001

**Table 4. Trends in the prevalence of current alcohol drinking**^1^

|  | | 2005 | 2007 | 2008 | 2009 | 2010 | 2011 | 2012 | 2013 | 2014 | 2015 | 2016 | 2017 | 2018 | Difference (2005 to 2018) | APC^3^ | APC and year of any significant change in trend slope | | |
| --- | --- | --- | --- | --- | --- | --- | --- | --- | --- | --- | --- | --- | --- | --- | --- | --- | --- | --- | --- |
|  |  |  |  |  |  |  |  |  |  |  |  |  |  |  |  |  | before | change year | after |
| Total (≥19 yr) | | 54.6 | 57.3 | 59.6 | 59.4 | 60.5 | 60.7 | 57.9 | 60.2 | 60.0 | 60.6 | 61.9 | 62.1 | 60.6 | 6.0 | **0.7*** | **2.8*** | 2008 | 0.3 |
| Men | | 72.6 | 73.5 | 74.7 | 75.8 | 77.8 | 77.7 | 73.6 | 75.3 | 74.4 | 75.2 | 75.3 | 74.0 | 70.5 | -2.1 | -0.1 | **1.3*** | 2010 | **-0.8*** |
| Age (yr) | 19-29 | 78.4 | 74.6 | 75.6 | 82.7 | 81.6 | 84.8 | 74.9 | 81.2 | 77.1 | 76.9 | 76.8 | 74.2 | 63.5 | -14.9 | -0.7 | 1.2 | 2011 | **-2.3*** |
|  | 30-39 | 77.8 | 79.9 | 79.9 | 78.1 | 84.9 | 81.9 | 79.0 | 78.5 | 79.0 | 80.0 | 82.6 | 78.6 | 75.2 | -2.6 | -0.1 | 1.3 | 2010 | -0.8 |
|  | 40-49 | 76.1 | 79.4 | 80.5 | 79.1 | 79.8 | 75.4 | 73.8 | 78.7 | 77.8 | 79.3 | 76.5 | 77.6 | 77.7 | 1.6 | 0.0 | 1.4 | 2008 | -0.3 |
|  | 50-59 | 71.5 | 75.8 | 74.1 | 75.1 | 78.1 | 81.2 | 75.6 | 74.9 | 72.1 | 74.0 | 74.1 | 73.4 | 71.5 | 0.0 | -0.2 | **1.5*** | 2011 | **-1.4*** |
|  | 60-69 | 60.7 | 58.3 | 65.6 | 66.4 | 66.6 | 70.6 | 64.8 | 67.4 | 65.0 | 65.0 | 68.2 | 69.9 | 69.8 | 9.1 | **0.8*** | 2.4 | 2009 | 0.4 |
|  | 70+ | 45.3 | 47.8 | 50.3 | 50.5 | 51.1 | 51.4 | 58.8 | 47.6 | 57.6 | 57.8 | 55.8 | 53.2 | 53.6 | 8.3 | **1.1*** | **2.2*** | 2015 | -2.7 |
| Household income^2^ | Low | 66.2 | 71.1 | 70.5 | 70.3 | 74.8 | 73.1 | 74.6 | 71.9 | 68.6 | 68.4 | 67.9 | 66.6 | 66.9 | 0.7 | -0.2 | **1.8*** | 2011 | **-1.8*** |
|  | Low-Middle | 71.3 | 68.6 | 74.7 | 75.8 | 75.7 | 75.0 | 72.2 | 71.6 | 72.5 | 70.8 | 74.2 | 74.0 | 70.8 | -0.5 | -0.1 | 1.4 | 2009 | -0.5 |
|  | Middle | 74.8 | 72.0 | 77.5 | 75.0 | 79.1 | 80.6 | 72.9 | 75.9 | 69.8 | 75.6 | 75.8 | 77.6 | 67.8 | -7.0 | -0.2 | 0.8 | 2011 | -1.0 |
|  | Middle-High | 74.8 | 75.9 | 75.1 | 78.0 | 80.4 | 80.6 | 71.4 | 78.3 | 78.8 | 80.3 | 78.7 | 72.5 | 70.4 | -4.4 | -0.1 | 0.4 | 2016 | -6.5 |
|  | High | 76.4 | 80.0 | 77.5 | 80.7 | 80.1 | 80.1 | 77.0 | 79.3 | 82.1 | 81.0 | 80.3 | 79.2 | 77.5 | 1.1 | 0.2 | **0.5*** | 2015 | -1.5 |
| Women | | 37.0 | 41.5 | 45.0 | 43.4 | 43.3 | 44.2 | 42.9 | 45.7 | 46.4 | 46.5 | 48.9 | 50.5 | 51.2 | 14.2 | **2.0*** | **5.0*** | 2008 | **1.6*** |
| Age (yr) | 19-29 | 51.8 | 63.1 | 63.8 | 55.4 | 52.1 | 60.2 | 57.7 | 62.3 | 60.3 | 56.6 | 64.1 | 66.3 | 65.7 | 13.9 | **1.3*** | 3.8 | 2008 | 0.8 |
|  | 30-39 | 41.2 | 43.5 | 50.3 | 50.1 | 49.1 | 52.6 | 48.0 | 56.4 | 53.9 | 50.6 | 55.5 | 58.6 | 60.0 | 18.8 | **2.3*** | 6.1 | 2008 | **1.8*** |
|  | 40-49 | 38.6 | 40.6 | 47.5 | 48.6 | 50.6 | 43.3 | 44.6 | 43.1 | 50.4 | 52.9 | 52.3 | 54.7 | 55.1 | 16.5 | **2.2*** | 6.1 | 2008 | **1.7*** |
|  | 50-59 | 28.6 | 36.0 | 34.4 | 33.9 | 36.4 | 34.9 | 35.7 | 39.1 | 36.6 | 42.1 | 42.0 | 39.0 | 41.4 | 12.8 | **2.4*** | 5.8 | 2008 | **2.0*** |
|  | 60-69 | 17.9 | 20.6 | 20.4 | 23.2 | 23.4 | 24.8 | 23.2 | 20.3 | 21.1 | 29.3 | 26.0 | 27.1 | 30.2 | 12.3 | **3.3*** | 2.3 | 2014 | 5.4 |
|  | 70+ | 14.5 | 10.4 | 15.0 | 13.8 | 15.5 | 14.4 | 16.3 | 15.3 | 20.3 | 13.8 | 13.5 | 16.6 | 12.5 | -2.0 | 0.2 | 2.8 | 2014 | -5.7 |
| Household income | Low | 36.6 | 40.2 | 44.6 | 41.7 | 44.0 | 44.2 | 41.6 | 40.8 | 44.0 | 44.6 | 46.6 | 50.4 | 49.6 | 13.0 | **1.9*** | **1.4*** | 2015 | 4.2 |
|  | Low-Middle | 37.9 | 43.7 | 46.4 | 42.0 | 42.4 | 41.9 | 42.9 | 42.6 | 47.3 | 45.1 | 45.7 | 49.8 | 56.4 | 18.5 | **2.2*** | **1.2*** | 2016 | 10.8 |
|  | Middle | 38.1 | 42.0 | 46.3 | 44.0 | 43.0 | 48.6 | 44.5 | 46.4 | 48.2 | 42.4 | 47.6 | 55.6 | 48.8 | 10.7 | **1.7*** | 5.0 | 2008 | 1.4 |
|  | Middle-high | 38.6 | 44.1 | 41.6 | 45.4 | 47.0 | 45.3 | 43.6 | 45.7 | 46.6 | 48.1 | 52.6 | 48.7 | 49.4 | 10.8 | **1.8*** | 3.6 | 2009 | **1.3*** |
|  | High | 34.1 | 38.3 | 46.3 | 43.7 | 39.6 | 42.5 | 43.5 | 53.3 | 45.8 | 52.0 | 50.8 | 48.1 | 51.6 | 17.5 | **2.7*** | 8.5 | 2008 | **1.8*** |

Values are presented as weighted %; Age-standardized prevalence was calculated using the 2005 population projections for Korea

^1^ Current alcohol drinking: percentage of adults who have had alcoholic drinks 1 or more times a month during the past year

^2^Calculated as monthly household income divided by the square root of the number of persons in the household, categorized into quantile by age and gender

^3^The annual percent change (APC) is significantly different from 0

* p < 0.001

**Table 5. Trends in the prevalence of binge drinking**^1^

|  | | 2005 | 2007 | 2008 | 2009 | 2010 | 2011 | 2012 | 2013 | 2014 | 2015 | 2016 | 2017 | 2018 | Difference (2005 to 2018) | APC^23^ | APC and year of any significant change in trend slope | | |
| --- | --- | --- | --- | --- | --- | --- | --- | --- | --- | --- | --- | --- | --- | --- | --- | --- | --- | --- | --- |
|  |  |  |  |  |  |  |  |  |  |  |  |  |  |  |  |  | before | change year | after |
| Total (≥19 yr) | | 36.2 | 37.1 | 39.5 | 40.0 | 39.9 | 39.0 | 38.0 | 37.4 | 37.5 | 38.7 | 39.3 | 39.0 | 38.9 | 2.7 | 0.2 | 2.9 | 2008 | -0.2 |
| Men | | 55.3 | 53.7 | 56.7 | 57.6 | 57.6 | 55.9 | 53.4 | 53.2 | 53.1 | 54.2 | 53.5 | 52.7 | 50.8 | -4.5 | **-0.7*** | 1.0 | 2009 | **-1.2*** |
| Age (yr) | 19-29 | 60.6 | 59.2 | 60.6 | 62.6 | 63.3 | 63.2 | 55.3 | 56.2 | 56.8 | 58.7 | 53.5 | 54.8 | 46.8 | -13.8 | **-1.4*** | 0.9 | 2010 | **-2.7*** |
|  | 30-39 | 62.1 | 62.1 | 63.1 | 62.2 | 66.8 | 65.1 | 60.5 | 59.4 | 60.8 | 64.1 | 62.8 | 57.9 | 55.9 | -6.2 | -0.6 | -0.1 | 2016 | -5.8 |
|  | 40-49 | 61.9 | 61.9 | 62.7 | 64.1 | 61.4 | 54.6 | 55.3 | 59.3 | 56.2 | 56.5 | 58.5 | 59.1 | 59.3 | -2.6 | **-0.6*** | **-1.0*** | 2015 | 1.6 |
|  | 50-59 | 52.3 | 49.1 | 60.4 | 58.5 | 57.5 | 59.0 | 57.9 | 54.5 | 52.4 | 51.3 | 53.0 | 52.5 | 51.5 | -0.8 | -0.7 | 5.0 | 2008 | **-1.5*** |
|  | 60-69 | 38.2 | 35.4 | 39.6 | 45.2 | 38.1 | 42.0 | 40.2 | 41.1 | 39.7 | 42.0 | 42.3 | 43.3 | 47.0 | 8.8 | **1.0*** | 0.4 | 2016 | 5.5 |
|  | 70+ | 25.0 | 17.8 | 20.7 | 22.3 | 24.6 | 19.4 | 27.4 | 19.0 | 26.4 | 22.2 | 23.9 | 23.0 | 23.9 | -1.1 | 0.5 | -2.8 | 2008 | 0.9 |
| Household income^2^ | Low | 51.7 | 57.0 | 55.5 | 54.0 | 55.0 | 50.9 | 55.1 | 49.8 | 51.5 | 49.0 | 47.2 | 48.3 | 47.8 | -3.9 | **-1.0*** | 2.4 | 2008 | **-1.7*** |
|  | Low-Middle | 51.9 | 51.6 | 56.7 | 60.7 | 56.0 | 54.0 | 52.6 | 48.7 | 52.5 | 50.5 | 54.8 | 55.8 | 52.6 | 0.7 | -0.3 | 2.6 | 2009 | -1.1 |
|  | Middle | 55.6 | 53.1 | 58.9 | 57.2 | 59.3 | 55.5 | 55.0 | 55.9 | 48.0 | 56.2 | 51.9 | 53.7 | 47.7 | -7.9 | **-0.8*** | 0.8 | 2010 | **-1.8*** |
|  | Middle-High | 59.6 | 53.7 | 58.5 | 58.3 | 59.9 | 58.7 | 46.6 | 56.3 | 54.6 | 59.1 | 57.2 | 51.7 | 51.5 | -8.1 | **-0.8*** | -0.5 | 2016 | -4.4 |
|  | High | 57.7 | 54.8 | 55.7 | 58.1 | 59.4 | 61.6 | 56.9 | 56.1 | 58.4 | 57.5 | 56.5 | 54.1 | 55.3 | -2.4 | -0.3 | 0.8 | 2011 | **-1.1*** |
| Women | | 17.2 | 20.5 | 22.3 | 22.2 | 22.1 | 22.1 | 22.9 | 21.9 | 22.5 | 23.3 | 25.0 | 25.0 | 26.9 | 9.7 | **2.4*** | **7.5*** | 2008 | **1.7*** |
| Age (yr) | 19-29 | 30.7 | 41.9 | 39.0 | 36.2 | 35.2 | 38.9 | 35.9 | 37.2 | 39.5 | 37.0 | 45.7 | 45.9 | 49.8 | 19.1 | **2.8*** | 1.7 | 2015 | 8.2 |
|  | 30-39 | 17.9 | 18.0 | 25.9 | 23.9 | 25.9 | 23.7 | 28.0 | 26.5 | 24.2 | 25.1 | 27.1 | 26.0 | 27.1 | 9.2 | **2.4*** | **12.1*** | 2008 | 0.9 |
|  | 40-49 | 17.8 | 17.7 | 23.1 | 23.8 | 22.2 | 21.0 | 21.4 | 18.5 | 21.8 | 23.6 | 23.0 | 22.8 | 26.0 | 8.2 | **1.5*** | 8.0 | 2008 | 0.6 |
|  | 50-59 | 10.7 | 15.2 | 10.9 | 14.7 | 16.3 | 15.9 | 17.9 | 16.5 | 15.3 | 17.3 | 16.5 | 16.2 | 17.9 | 7.2 | **3.0*** | **7.3*** | 2011 | 0.3 |
|  | 60-69 | 3.9 | 5.5 | 4.8 | 7.9 | 5.4 | 6.6 | 7.1 | 6.2 | 6.1 | 10.0 | 7.4 | 9.6 | 8.5 | 4.6 | **5.5*** | 12.8 | 2009 | **4.0*** |
|  | 70+ | 1.8 | 1.0 | 2.0 | 1.4 | 2.2 | 1.6 | 1.8 | 2.0 | 2.7 | 3.4 | 1.0 | 2.0 | 1.0 | -0.8 | 2.2 | **7.1*** | 2015 | -24.5 |
| Household income | Low | 17.4 | 23.9 | 27.9 | 24.7 | 25.8 | 25.5 | 25.0 | 20.9 | 23.4 | 26.6 | 27.4 | 29.8 | 29.3 | 11.9 | **2.4*** | 12.1 | 2008 | 1.1 |
|  | Low-Middle | 19.6 | 21.5 | 23.6 | 22.1 | 20.5 | 21.4 | 23.6 | 23.0 | 23.7 | 23.2 | 24.7 | 25.6 | 29.9 | 10.3 | **2.5*** | **1.6*** | 2016 | 10.5 |
|  | Middle | 17.1 | 19.7 | 22.2 | 21.5 | 22.4 | 24.0 | 23.6 | 22.1 | 20.0 | 24.0 | 26.9 | 27.0 | 26.2 | 9.1 | **2.7*** | 7.7 | 2008 | **2.1*** |
|  | Middle-High | 18.0 | 21.9 | 17.8 | 23.8 | 22.5 | 20.6 | 22.6 | 20.7 | 22.6 | 20.5 | 24.2 | 21.3 | 24.9 | 6.9 | **1.4*** | 5.0 | 2009 | 0.5 |
|  | High | 14.3 | 14.3 | 20.2 | 18.2 | 18.2 | 19.3 | 19.6 | 23.0 | 22.8 | 22.4 | 20.9 | 21.1 | 24.1 | 9.8 | **3.4*** | 9.9 | 2008 | **2.3*** |

Values are presented as weighted %; Age-standardized prevalence was calculated using the 2005 population projections for Korea

^1^Binge drinking: percentage of adults who have drunk ≥ 7 (men) or ≥ 5 (women) alcoholic drinks more than once a week during the past year

^2^Calculated as monthly household income divided by the square root of the number of persons in the household, categorized into quantile by age and gender

^3^The annual percent change (APC) is significantly different from 0

* p < 0.001

**Table 6. Trends in the prevalence of aerobic physical activity**^1^

|  | | 2007 | 2008 | 2009 | 2010 | 2011 | 2012 | 2013 | 2014 | 2015 | 2016 | 2017 | 2018 | Difference (2007 to 2018) | APC^3^ | APC and year of any significant change in trend slope | | |
| --- | --- | --- | --- | --- | --- | --- | --- | --- | --- | --- | --- | --- | --- | --- | --- | --- | --- | --- |
|  |  |  |  |  |  |  |  |  |  |  |  |  |  |  |  | before | change year | after |
| Total (≥19 yr) | | 72.5 | 75.5 | 76.4 | 71.8 | 67.8 | 68.1 | 70.3 | 58.3 | 52.7 | 49.4 | 48.5 | 47.6 | -24.9 | **-4.5*** | **-2.5*** | 2013 | **-7.9*** |
| Men | | 76.3 | 79.2 | 80.4 | 75.7 | 72.1 | 73.1 | 76.8 | 62.0 | 55.8 | 52.5 | 50.6 | 51.0 | -25.3 | **-4.2*** | **-1.9*** | 2013 | **-8.3*** |
| Age (yr) | 19-29 | 83.6 | 85.8 | 87.6 | 81.9 | 77.2 | 79.7 | 85.9 | 79.8 | 72.1 | 66.9 | 67.0 | 69.7 | -13.9 | **-2.2*** | -0.8 | 2013 | **-4.6*** |
|  | 30-39 | 72.9 | 75.5 | 75.2 | 74.6 | 69.0 | 69.9 | 74.0 | 58.2 | 54.3 | 50.7 | 51.3 | 55.3 | -17.6 | **-3.6*** | **-1.9*** | 2013 | **-6.7*** |
|  | 40-49 | 73.5 | 75.0 | 79.4 | 74.4 | 70.7 | 75.1 | 76.4 | 57.5 | 55.1 | 50.7 | 48.4 | 48.8 | -24.7 | **-4.2*** | -1.1 | 2013 | **-9.3*** |
|  | 50-59 | 80.4 | 80.7 | 82.4 | 74.5 | 72.6 | 71.8 | 73.0 | 60.2 | 48.3 | 44.7 | 45.9 | 36.5 | -43.9 | **-6.1*** | **-3.0*** | 2013 | **-12.1*** |
|  | 60-69 | 79.1 | 83.1 | 81.4 | 76.3 | 72.0 | 71.3 | 73.2 | 53.7 | 46.6 | 50.9 | 34.4 | 40.8 | -38.3 | **-6.4*** | **-3.5*** | 2013 | **-12.0*** |
|  | 70+ | 61.4 | 75.0 | 71.9 | 64.8 | 69.7 | 61.5 | 70.6 | 48.2 | 38.3 | 35.6 | 34.8 | 27.7 | -33.7 | **-6.6*** | -1.5 | 2013 | **-17.1*** |
| Household income^2^ | Low | 72.3 | 79.4 | 77.8 | 72.6 | 72.2 | 71.1 | 74.1 | 59.9 | 55.6 | 43.1 | 49.7 | 48.4 | -23.9 | **-4.6*** | -2.1 | 2013 | **-8.6*** |
|  | Low-Middle | 70.0 | 78.7 | 78.2 | 75.2 | 71.4 | 66.6 | 70.6 | 60.4 | 56.5 | 52.7 | 48.9 | 48.6 | -21.4 | **-4.5*** | 3.9 | 2009 | **-5.4*** |
|  | Middle | 79.3 | 76.5 | 82.5 | 76.6 | 70.2 | 72.5 | 77.1 | 62.7 | 52.5 | 51.7 | 51.1 | 46.6 | -32.7 | **-4.4*** | -1.7 | 2013 | **-9.4*** |
|  | Middle-High | 77.4 | 80.4 | 81.2 | 74.2 | 73.7 | 78.3 | 77.1 | 65.4 | 52.7 | 54.5 | 51.2 | 54.6 | -22.8 | **-4.0*** | -1.7 | 2013 | **-7.9*** |
|  | High | 82.2 | 82.2 | 82.2 | 80.8 | 73.8 | 78.0 | 85.1 | 61.5 | 61.5 | 60.4 | 51.9 | 57.6 | -24.6 | **-3.4*** | -0.8 | 2013 | **-7.9*** |
| Women | | 68.9 | 72.1 | 72.5 | 68.0 | 63.8 | 63.3 | 64.0 | 54.7 | 49.8 | 46.4 | 46.6 | 44.0 | -24.9 | **-4.7*** | 2.5 | 2009 | **-5.5*** |
| Age (yr) | 19-29 | 69.7 | 74.2 | 75.7 | 69.4 | 68.2 | 72.5 | 69.2 | 64.1 | 60.8 | 56.4 | 63.9 | 57.1 | -12.6 | **-2.4*** | 2.4 | 2009 | **-2.8*** |
|  | 30-39 | 64.2 | 70.1 | 68.0 | 68.7 | 61.8 | 63.9 | 61.1 | 57.8 | 48.3 | 47.4 | 43.6 | 45.8 | -18.4 | **-4.2*** | 0.9 | 2010 | **-5.6*** |
|  | 40-49 | 75.5 | 75.0 | 74.8 | 69.4 | 65.8 | 62.8 | 68.6 | 56.9 | 54.0 | 45.0 | 46.6 | 42.8 | -32.7 | **-5.0*** | **-3.1*** | 2013 | **-8.4*** |
|  | 50-59 | 73.3 | 76.8 | 77.2 | 71.0 | 67.1 | 61.3 | 66.1 | 52.6 | 44.2 | 46.0 | 43.0 | 39.2 | -34.1 | **-6.2*** | **-4.2*** | 2013 | **-9.6*** |
|  | 60-69 | 68.0 | 68.9 | 72.9 | 68.7 | 63.2 | 59.6 | 59.8 | 42.7 | 44.9 | 41.0 | 37.1 | 36.6 | -31.4 | **-6.4*** | 5.0 | 2009 | **-7.7*** |
|  | 70+ | 53.8 | 58.9 | 59.9 | 50.7 | 44.6 | 43.8 | 45.2 | 29.1 | 24.2 | 23.9 | 22.0 | 20.5 | -33.3 | **-10.1*** | 4.5 | 2009 | **-11.7*** |
| Household income | Low | 68.6 | 67.9 | 72.9 | 70.1 | 60.4 | 61.6 | 58.1 | 51.9 | 48.7 | 42.5 | 45.1 | 40.8 | -27.8 | **-5.4*** | 4.7 | 2009 | **-6.3*** |
|  | Low-Middle | 65.6 | 71.5 | 72.7 | 67.2 | 65.1 | 62.1 | 65.1 | 56.9 | 48.3 | 41.6 | 45.6 | 43.3 | -22.3 | **-4.8*** | -2.5 | 2013 | **-8.6*** |
|  | Middle | 72.1 | 76.5 | 72.7 | 68.8 | 62.4 | 65.4 | 65.0 | 52.1 | 50.0 | 45.8 | 44.7 | 44.2 | -27.9 | **-5.3*** | **-3.9*** | 2013 | **-7.8*** |
|  | Middle-High | 69.7 | 74.2 | 73.2 | 67.0 | 66.0 | 61.2 | 64.3 | 55.3 | 47.6 | 52.8 | 46.2 | 45.4 | -24.3 | **-4.6*** | 0.7 | 2009 | **-5.3*** |
|  | High | 69.2 | 71.5 | 70.9 | 66.8 | 65.5 | 66.8 | 68.0 | 56.9 | 53.7 | 49.4 | 51.3 | 46.7 | -22.5 | **-3.7*** | **-1.6*** | 2013 | **-6.9*** |

Values are presented as weighted %; Age-standardized prevalence was calculated using the 2005 population projections for Korea

^1^Aerobic physical activity: percentage of adults who have performed 150 minutes of moderate-intensity physical activity or 75 minutes of vigorous-intensity physical activity or an equivalent combination of moderate- and vigorous-intensity physical activity in a typical week

^2^Calculated as monthly household income divided by the square root of the number of persons in the household, categorized into quantile by age and gender

^3^The annual percent change (APC) is significantly different from 0

* p < 0.001

**Table 7. Trends in the prevalence of healthy behaviors practice**^1^

|  | | 2007 | 2008 | 2009 | 2010 | 2011 | 2012 | 2013 | 2014 | 2015 | 2016 | 2017 | 2018 | Difference (2007 to 2018) | APC^3^ | APC and year of any significant change in trend slope | | |
| --- | --- | --- | --- | --- | --- | --- | --- | --- | --- | --- | --- | --- | --- | --- | --- | --- | --- | --- |
|  |  |  |  |  |  |  |  |  |  |  |  |  |  |  |  | before | change year | after |
| Total (≥19 yr) | | 25.1 | 25.1 | 25.1 | 23.7 | 21.4 | 23.8 | 22.6 | 19.2 | 17.4 | 15.2 | 14.8 | 14.5 | -10.6 | **-5.3*** | **-2.6*** | 2013 | **-9.5*** |
| Men | | 12.5 | 13.1 | 12.3 | 11.1 | 10.2 | 14.4 | 13.1 | 10.9 | 10.5 | 9.4 | 8.8 | 10.7 | -1.8 | **-2.5*** | -0.4 | 2013 | -5.3 |
| Age (yr) | 19-29 | 13.5 | 13.5 | 10.9 | 11.7 | 7.6 | 17.9 | 10.3 | 12.3 | 13.3 | 11.4 | 12.1 | 17.4 | 3.9 | 1.5 | -0.6 | 2016 | 17.1 |
|  | 30-39 | 6.0 | 8.7 | 8.2 | 5.3 | 5.0 | 10.9 | 12.0 | 9.7 | 8.7 | 6.6 | 7.4 | 10.0 | 4.0 | 1.7 | 6.3 | 2013 | -4.0 |
|  | 40-49 | 9.2 | 9.0 | 9.0 | 11.0 | 11.6 | 10.8 | 10.5 | 8.0 | 6.9 | 9.2 | 6.6 | 8.1 | -1.1 | -2.5 | 7.5 | 2011 | **-6.3*** |
|  | 50-59 | 14.5 | 15.3 | 15.6 | 9.4 | 9.1 | 12.2 | 12.6 | 12.3 | 10.7 | 8.8 | 8.1 | 7.2 | -7.3 | **-5.8*** | -4.2 | 2015 | -12.7 |
|  | 60-69 | 24.1 | 20.3 | 17.5 | 16.9 | 16.1 | 22.0 | 16.4 | 11.2 | 13.2 | 10.9 | 7.1 | 9.2 | -14.9 | **-7.7*** | -2.1 | 2012 | **-12.5*** |
|  | 70+ | 20.9 | 24.8 | 27.0 | 24.0 | 24.9 | 19.9 | 29.6 | 16.3 | 14.7 | 12.6 | 13.0 | 8.6 | -12.3 | **-8.1*** | -0.2 | 2013 | **-17.9*** |
| Household income^2^ | Low | 12.8 | 13.4 | 13.4 | 13.1 | 10.3 | 12.0 | 13.9 | 11.9 | 12.0 | 12.0 | 9.9 | 9.4 | -3.4 | **-2.4*** | -1.3 | 2016 | -11.8 |
|  | Low-Middle | 15.5 | 11.8 | 10.8 | 11.5 | 12.4 | 12.3 | 14.0 | 9.7 | 14.3 | 8.5 | 8.5 | 9.2 | -6.3 | **-2.8*** | -0.8 | 2015 | -10.4 |
|  | Middle | 13.7 | 11.6 | 13.7 | 10.5 | 9.1 | 16.3 | 13.1 | 12.1 | 10.0 | 8.7 | 8.3 | 11.2 | -2.5 | -2.5 | 0.3 | 2013 | -6.3 |
|  | Middle-High | 10.9 | 14.6 | 13.0 | 9.9 | 7.8 | 14.9 | 12.3 | 12.2 | 8.2 | 8.4 | 9.7 | 14.2 | 3.3 | -1.1 | -3.9 | 2016 | 19.8 |
|  | High | 8.3 | 13.4 | 10.2 | 10.6 | 11.0 | 16.6 | 11.8 | 8.8 | 7.8 | 9.3 | 7.2 | 9.3 | 1.0 | -3.1 | 5.3 | 2012 | **-9.1*** |
| women | | 37.3 | 36.8 | 37.8 | 36.2 | 32.5 | 32.9 | 31.9 | 27.1 | 24.2 | 21.0 | 20.9 | 18.3 | -19.0 | **-6.4*** | **-3.5*** | 2013 | **-10.7*** |
| Age (yr) | 19-29 | 21.2 | 24.7 | 30.5 | 30.1 | 23.1 | 25.6 | 22.9 | 23.9 | 23.6 | 19.6 | 18.9 | 15.9 | -5.3 | **-3.8*** | 20.7 | 2009 | **-5.8*** |
|  | 30-39 | 35.4 | 30.9 | 29.8 | 34.4 | 25.1 | 32.4 | 24.4 | 25.3 | 23.3 | 18.8 | 18.4 | 16.1 | -19.3 | **-5.9*** | -2.2 | 2012 | **-9.3*** |
|  | 40-49 | 41.7 | 38.4 | 36.8 | 32.8 | 37.7 | 32.2 | 34.4 | 28.1 | 24.2 | 18.0 | 21.6 | 17.9 | -23.8 | **-6.8*** | -3.5 | 2013 | **-12.0*** |
|  | 50-59 | 46.6 | 48.8 | 48.3 | 43.5 | 41.6 | 36.5 | 41.9 | 31.2 | 24.3 | 25.4 | 25.6 | 21.6 | -25.0 | **-7.3*** | **-4.7*** | 2013 | **-11.2*** |
|  | 60-69 | 49.5 | 51.8 | 53.5 | 49.6 | 45.9 | 44.5 | 46.5 | 33.0 | 30.9 | 31.1 | 25.7 | 26.3 | -23.2 | **-6.8*** | **-3.9*** | 2013 | **-11.1*** |
|  | 70+ | 46.2 | 45.3 | 47.2 | 38.9 | 34.2 | 36.1 | 36.5 | 24.1 | 19.8 | 19.2 | 18.0 | 16.1 | -30.1 | **-9.7*** | **-6.6*** | 2013 | **-14.7*** |
| Household income | Low | 33.4 | 34.3 | 38.6 | 35.5 | 29.3 | 31.1 | 32.0 | 26.4 | 24.5 | 19.9 | 19.9 | 18.4 | -15.0 | **-6.1*** | 8.1 | 2009 | **-7.5*** |
|  | Low-Middle | 33.6 | 35.8 | 38.1 | 37.0 | 34.7 | 32.7 | 35.6 | 27.5 | 24.9 | 20.4 | 20.6 | 14.0 | -19.6 | **-6.6*** | -1.1 | 2013 | **-15.0*** |
|  | Middle | 43.4 | 37.1 | 37.6 | 37.3 | 30.5 | 32.4 | 29.8 | 24.9 | 25.6 | 19.1 | 17.7 | 21.7 | -21.7 | **-6.9*** | -4.7 | 2010 | **-7.5*** |
|  | Middle-High | 36.0 | 40.5 | 35.3 | 33.5 | 32.7 | 32.3 | 32.6 | 26.9 | 23.3 | 22.3 | 21.8 | 17.2 | -18.8 | **-6.2*** | **-3.7*** | 2013 | **-10.4*** |
|  | High | 40.7 | 36.3 | 38.7 | 37.4 | 34.5 | 35.7 | 29.5 | 29.5 | 22.6 | 23.4 | 25.1 | 20.3 | -20.4 | **-5.7*** | -3.2 | 2012 | **-8.2*** |

Values are presented as weighted %; Age-standardized prevalence was calculated using the 2005 population projections for Korea

^1^Healthy behaviors Practice: percentage of adults who met the guidelines for cigarette smoking, alcohol drinking, and physical activity

^2^Calculated as monthly household income divided by the square root of the number of persons in the household, categorized into quantile by age and gender

^3^The annual percent change (APC) is significantly different from 0

* p < 0.001
